# Current Computational Approaches for the Discovery of Novel Anticancer Agents Targeting VEGFR and SIRT Signaling Pathways

**DOI:** 10.3390/pharmaceutics18020273

**Published:** 2026-02-22

**Authors:** Aleksandra Ilic, Selma Zukic, Slavica Oljacic, Uko Maran, Katarina Nikolic, Marija Popovic-Nikolic

**Affiliations:** 1 Department of Pharmaceutical Chemistry, Faculty of Pharmacy, University of Belgrade, Vojvode Stepe, 450, 11221 Belgrade, Serbia; aleksandra.ilic@pharmacy.bg.ac.rs (A.I.); slavica.oljacic@pharmacy.bg.ac.rs (S.O.); katarina.nikolic@pharmacy.bg.ac.rs (K.N.); 2 Institute of Chemistry, University of Tartu, Ravila Street 14a, 50411 Tartu, Estonia; uko.maran@ut.ee

**Keywords:** VEGFR, SIRT, multifactorial diseases, dual-inhibitors, QSAR, machine learning, SBDD

## Abstract

Numerous scientific studies highlight the crucial role of common genetic and epigenetic factors in the development and progression of cancer. To deepen our understanding of how different VEGFR and epigenetic pathways interact in carcinogenesis, the current review examines novel therapeutic agents that target various molecular mechanisms involved in this complex disease. Growing evidence from scientific studies suggests that VEGFR and epigenetic signaling pathways contribute to complex pathophysiological changes in cancer. Therefore, simultaneously targeting VEGFR and epigenetic factors, such as sirtuins, by developing dual inhibitors could provide more individualized therapeutic approaches with safer and more effective outcomes. In this context, Computer-Aided Drug Design (CADD) offers a comprehensive suite of bioinformatic, chemoinformatic, and chemometric approaches to design novel chemotypes of epigenetic dual-target inhibitors. This facilitates the efficient discovery of new drug candidates, enabling innovative treatments for these multifactorial diseases. The review also explores the detailed anticancer mechanisms by which VEGFR, SIRT, and dual-target inhibitors modify metastatic and tumorigenic properties, affect the tumor microenvironment, and regulate the immune response.

## 1. Introduction

This review briefly analyses and compares computational approaches that have been successfully applied to the development of dual inhibitors for cancer therapy, targeting the vascular endothelial growth factor receptor (VEGFR) and sirtuin signaling pathways (SIRT). Computer-Aided Drug Design (CADD) enables efficient discovery of novel multi-target chemotherapeutic agents. By effectively applying and combining molecular docking, virtual screening, quantitative structure–activity relationship (QSAR) and other computational strategies, CADD contributes to the development of novel dual chemotypes that selectively target both VEGFR and sirtuin signaling pathways. This review highlights the role of computational approaches in the design, discovery and development of dual inhibitors, which have contributed to the improvement of innovative anticancer therapeutic strategies. It also emphasises the potential of computational approaches to transform traditional cancer treatment methods and provide us with better insight into precision medicine and more effective therapeutic interventions for cancer patients.

### 1.1. VEGFR and Epigenetic SIRT Pathways

Knowledge of the complex pathophysiological mechanisms of tumorigenesis is essential for the development of modern therapeutic strategies and the improvement of clinical applications. Tumor growth and progression result not from isolated molecular changes, but from an extensive network of interconnected signaling pathways that regulate cellular proliferation, differentiation, migration, and survival [1,2]. Key regulators of tumorigenesis include VEGFR, epigenetic modification systems, and the family of NAD^+^-dependent histone deacetylases known as sirtuins (SIRTs) [3,4,5,6,7].

The VEGFR signaling pathway plays a central role in angiogenesis, the biological process that leads to the formation of capillary blood vessels supplying tumor cells with oxygen and nutrients. After VEGFR signaling is initiated on endothelial cells, a cascade of intracellular signals begins, involving the phosphoinositide 3-kinase/protein kinase B (PI3K/AKT) and mitogen-activated protein kinase/extracellular signal-regulated kinase (MAPK/ERK) pathways, which promote cell proliferation, migration, and vascular remodeling. In addition, VEGFR signaling contributes to the creation of an immunosuppressive tumor microenvironment that supports tumor survival and further development [5,8,9,10,11]. Epigenetic modifications such as DNA methylation and post-translational histone modifications (acetylation, methylation, phosphorylation) flexibly control gene expression without altering the underlying DNA sequence [3,7]. These reversible changes enable tumor cells to adapt to stressful conditions such as hypoxia and therapeutic pressure. Epigenetic regulation also influences the expression of VEGFR and other angiogenesis-related factors, further complicating the signaling network and hindering effective therapeutic intervention [3,6,7,12,13,14,15].

In this complex system, sirtuins—particularly SIRT2—establish a link between epigenetic regulation and the cell’s response to metabolic processes. SIRT2 is an NAD^+^-dependent histone and non-histone deacetylase that controls the activity of various proteins regulating the cell cycle, apoptosis, metabolism, and cell death. By regulating histone acetylation, SIRT2 influences chromatin structure and accessibility to transcription factors, including those that control genes related to angiogenesis and cell proliferation [3,5,7,12,15,16,17,18,19,20,21]. In addition, SIRT2 directly deacetylates key transcription factors and signaling molecules associated with VEGFR signaling pathways, coordinating cellular responses to external stimuli. New evidence suggests a reciprocal regulatory relationship between VEGFR signaling and SIRT2 activity [22]. Several studies provide a strong mechanistic rationale for the interaction between sirtuins and VEGFR signaling [16,18], highlighting that sirtuins regulate key processes in endothelial cells, modulate VEGF/VEGFR signaling, and influence angiogenic responses in cancer. These findings support the concept that simultaneous modulation of VEGFR and SIRT pathways may have synergistic effects on angiogenesis and tumor progression. SIRT2 inhibitors such as RK-9123016, NPD11033, and fluvastatin sodium have demonstrated potential in inhibiting tumor cell proliferation by downregulating c-Myc expression and increasing the acetylation of target proteins, which collectively reduce cell growth and suppress metastasis [8,23]. Wang et al. showed that combining a SIRT2 inhibitor with sorafenib—a clinically approved multi-kinase inhibitor targeting receptor tyrosine kinases (e.g., VEGFR-1, VEGFR-2, VEGFR-3, and PDGFR) and serine/threonine kinases (e.g., RAF1 and B-Raf)—resulted in a synergistic antitumor effect in MCF-7 cells compared to monotherapy. The existence of synergistic activity was demonstrated through in vitro and in vivo studies involving MCF-7 xenograft mouse models [8]. Based on these studies, the therapeutic potential of SIRT2 targets has been highlighted, justifying further investigation of this target in dual-targeted therapies for various cancer types.

Although VEGFR signaling pathways are a central target in antiangiogenic therapy, the clinical efficacy of this approach is often limited by resistance mechanisms, including metabolic reprogramming, hypoxia-induced responses [5,8,9,10,11], and epigenetic plasticity [3,6,7,12,13,14,15]. Sirtuins, as regulators of stress responses and chromatin dynamics, are involved in several of these adaptive processes and have also been linked to the modulation of angiogenic signaling and endothelial function [8,22]. In this context, considering VEGFR and sirtuins within the same framework is based on the idea that simultaneous modulation of angiogenic pathways and epigenetic regulators may help overcome mechanisms that limit the long-term benefit of current therapies. This biological overlap may provide complementarity and represents a direction for future research.

Tyrosine kinases (TKs) are enzymes with essential functions in regulating various cellular processes, including growth, differentiation, and proliferation [8]. Among them, receptor tyrosine kinases (RTKs) are particularly important, as their activation initiates downstream signaling pathways that regulate tumor cell proliferation and angiogenesis [9]. VEGFR activation can modulate the expression and function of SIRT2 via downstream signaling cascades, while SIRT2 epigenetically regulates genes involved in VEGFR-mediated angiogenesis and tumor progression. This interplay enables tumor cells to establish adaptive mechanisms that promote growth, invasion, and evasion of the immune system. Given this complex interplay, targeting individual molecular signaling pathways often proves insufficient for sustained therapeutic efficacy. Integrated approaches that simultaneously modulate VEGFR signaling and epigenetic regulation via SIRT enzymes represent promising strategies to overcome tumor resistance and achieve better disease control [11,22]. Dual inhibition of VEGFR and sirtuin pathways may improve anticancer efficacy by targeting both tumor vascular support and epigenetic adaptability. While VEGFR inhibition suppresses angiogenesis and limits nutrient supply, sirtuin modulation affects chromatin regulation and transcriptional processes involved in proliferation, stress response, and adaptation to hypoxia. As these processes are interconnected, their simultaneous disruption may impair compensatory mechanisms that reduce the efficacy of single-target therapies. In addition, modulation of these pathways may indirectly influence the immune response, as abnormal vascular signaling and epigenetic remodeling promote conditions that attenuate immune recognition of malignant cells. This mechanism suggests that targeting multiple targets may provide synergistic benefits over modulating a single pathway, simultaneously addressing angiogenic, epigenetic, and immunological aspects of tumor growth. The development of dual inhibitors targeting these key molecular nodes holds great potential to advance cancer therapy [24,25].

### 1.2. Polypharmacology and Multitarget Approach in Cancer Research

Traditional drug discovery methods have largely followed the ‘one drug–one target’ paradigm. This strategy has led to the development of many highly potent, selective drugs designed to act on specific biological targets. While this approach remains important for diseases with clearly defined mechanisms, etiology, and pathophysiology, it also contributes to the high rate of clinical trial failures, mainly due to its limited ability to predict adverse side effects and toxicity [26]. For multifactorial diseases such as cancer [26,27,28], neurodegenerative diseases [29,30,31], infectious diseases [32], metabolic syndrome [33], or cardiovascular diseases [34], which are driven by highly complex mechanisms, polypharmacology has emerged as a powerful and promising alternative paradigm that enables the development of various therapeutic agents for urgent medical needs. According to the National Library of Medicine, polypharmacology is a concept in drug discovery based on the design or use of compounds that exert effects on multiple targets or disease mechanisms [35]. The benefits of polypharmacology can be broadly classified into two types. The first involves off-target effects, where inhibition of a secondary target, rather than the primary one, produces therapeutic activity in a different indication, enabling drug repurposing. The second, more complex type, involves synergistic or additive effects from simultaneous inhibition of multiple targets within the same indication [36].

The Multitargeted Ligand (MTDL) approach offers many advantages, representing a significant alternative to combination therapies while reducing the need for the simultaneous use of multiple drugs [37]. This can improve patient compliance and reduce pharmacokinetic complexity, adverse effects, and drug interactions. Well-designed and optimally balanced MTDLs may also enhance therapeutic efficacy, allow for lower dosages, and provide economic benefits, as a single MTDL typically requires fewer clinical trials compared to multiple specific drugs [24,37].

In the development of polypharmacology, the first multitargeted ligands were discovered by chance, while modern medicinal chemistry increasingly emphasizes the rational design of polypharmacological agents. This approach involves identifying appropriate combinations of targets for MTDLs and designing, identifying, and optimizing ligands capable of simultaneously controlling multiple targets. Selecting suitable combinations of targets that produce additive or synergistic effects is a crucial and challenging task that requires a deep understanding of disease mechanisms. The extensive use of multi-kinase inhibitors (MKIs) in cancer therapy, which involves simultaneously targeting multiple signaling pathways in cell proliferation, angiogenesis, and migration, is an illustrative example of synergism based on polypharmacology. Kinase inhibitors have significant potential for multitargeting due to the highly conserved adenosine triphosphate (ATP) binding site shared across all kinases. Cabozantinib is an MKI that targets MET, vascular endothelial growth factor receptor 2 (VEGFR-2), and rearranged during transfection (RET) across multiple cancer types [38]. It is essential that selected targets are compatible in terms of tissue localization and ligand binding properties to minimize complexity during drug development [39]. Several strategies have been proposed to validate target combinations, including approaches based on clinical observations, phenotypic screening, and in silico techniques.

Antipsychotics and anticancer drugs are prime examples of multitarget drug discovery driven by clinical observations [40,41]. These drugs are often prescribed in combination therapies that act on distinct targets. Although drug combinations have shown many disadvantages in practice, confirmed therapeutic benefits have incentivized the rational design of multitargeted agents based on target combinations previously validated through combination therapies. For example, the simultaneous targeting of vascular endothelial growth factor (VEGF) and PD-1/PD-L1 pathways in oncology has emerged from clinical evidence highlighting the interdependence of tumor angiogenesis and immune evasion in cancer progression [42].

Although not the subject of this paper, it is important to note that other approaches for identifying effective target combinations exist, such as phenotypic screening. In the search for combinations of compounds that exhibit synergistic effects, researchers can screen cell, tissue, and animal models [43].

### 1.3. In Silico Identification of Multitarget-Directed Ligands of VEGFR and SIRT

In silico techniques have been successfully used to identify promising target combinations. Various computational methods, including machine learning, statistical modeling, pathway and network analysis, and integrated in silico/in vitro approaches, have been described for predicting effective combinations of targets and drugs [40]. Although in silico predictions can yield promising results, they require subsequent experimental validation to confirm their relevance and accuracy. This validation process usually involves biochemical assays, cell-based activity studies, and in vivo tumor models, complemented by pharmacokinetic and toxicity evaluations to confirm target engagement and translational potential.

For example, Djokovic and coauthors investigated the potential of using drug sensitivity data alongside basal gene expression profiles from pancreatic cell lines to predict combinatorial treatment options for histone deacetylase inhibitors (HDACis). Their findings highlight the sphingolipid signaling pathway and its downstream effectors as promising targets for combination therapy with HDACi or for the future development of multitarget therapeutics [44]. Based on these findings, Beljkas and coauthors reported the rational design, synthesis, and biological evaluation of first-in-class HDAC/ROCK multitarget inhibitors in models of pancreatic ductal adenocarcinoma (PDAC) and triple-negative breast cancer (TNBC) [27].

As already highlighted, the rational design of polypharmacological compounds also includes the identification and optimization of ligands directed toward multiple targets. In their chemical structure, these compounds combine two or more pharmacophores, which enables interaction with multiple biological targets. Depending on the degree of pharmacophore overlap, MTDLs are classified as linked, fused, or merged [24].

Linked MTDLs consist of distinct pharmacophore elements for each target, separated by a linker group that is not present in either of the original selective ligands. The linker can be designed to be metabolically stable, maintaining the integrity of the compound, or cleavable, allowing metabolism to release two active ligands that independently interact with their respective targets. The chemistry of the linker—its design and attachment—plays a very important role and largely determines the properties and behavior of the drug. Using these strategies, two potent compounds can be combined without excessive structural optimization. However, this approach often results in large molecules with low bioavailability and poor drug-like properties. Although there have been some shortcomings, this concept has attracted considerable attention in the development of antibody-drug conjugates, where the antibody primarily serves as a carrier to deliver a pharmacologically active compound to the target site of action [45].

If the compounds are formed by direct fusion of pharmacophores, they are fused MTDLs and often show partial overlap of their initial structures. As with linked MTDLs, where optimizing the linker attachment site is critical, the site and mode of fusion in fused MTDLs must be carefully assessed and refined [39]. This design strategy generally results in compounds with slightly lower molecular weights compared to their linked counterparts. However, they often retain high lipophilicity and may exhibit unfavorable pharmacokinetic profiles. Despite these challenges, the fused MTDL approach has shown promise, particularly in the development of multitarget compounds with anticonvulsant [46] and anticancer activity [27].

Merged MTDLs are the most common type of small molecules designed to modulate multiple targets. They feature the highest degree of pharmacophore overlap, which typically results in lower molecular weight compounds with improved pharmacokinetic properties. Several successful projects have produced merged multi-target ligands through the rational and systematic combination of common scaffolds and substructures found in selective ligands for the respective targets. Notable examples of this approach can be found in the design of MTDLs for the treatment of neurodegenerative diseases [47], and dual aromatase-steroid sulfatase inhibitors [48] can be highlighted as successful applications of this approach.

The presence of MTDLs in modern pharmacology is evident from the number of drugs approved for use. Ryszkiewicz et al. [49] analyzed drugs approved in Germany before 2022. Ten of these were identified as multitargeting, including seven antitumor agents, one antidepressant, one hypnotic, and one drug indicated for eye disease. Among the approved drugs, three multi-kinase inhibitors (MKIs) were identified: axitinib, cabozantinib, and sunitinib, all of which, among other targets, act on VEGFR-2 and are used in the treatment of various types of cancer [49].

**Figure 1:**
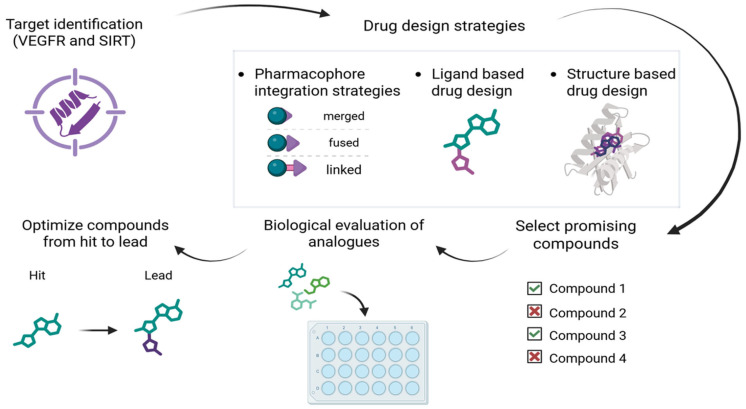
CADD/Biochemistry workflow for development of novel VEGFR/SIRT multitarget inhibitors.

Despite some remaining challenges, significant advances in computational methods, particularly in artificial intelligence, have made the multitarget-directed approach highly promising for future therapeutic innovation (Figure 1). Computer-aided drug design (CADD) has transformed the landscape of MTDLs drug discovery and development [50]. Based on the underlying theoretical framework, CADD approaches can be broadly classified into two categories: ligand-based drug design (LBDD) and structure-based drug design (SBDD). Prediction of drug polypharmacology using ligand-based methods relies primarily on chemical structures and their bioactivities. The LBDD approach includes ligand-based virtual screening (LBVS), similarity searching, QSAR modeling, and pharmacophore generation. Target-based polypharmacology relies on the availability of high-resolution crystal structures of target proteins and encompasses strategies such as structure-based virtual screening (SBVS), molecular docking, and molecular dynamics simulations [50]. In this context, current in silico techniques such as machine learning-based QSAR modeling, pharmacophore analysis, molecular docking, and virtual screening have contributed to the identification of promising dual-target lead structures with predicted high binding affinity and selectivity. By enabling early-stage identification of potential candidates before synthesis and biological testing, these strategies reduce reliance on extensive experimental procedures and provide a more focused and rational basis for subsequent lead optimization. Over time, there have been many successful applications of CADD in the design of new drug compounds, which have become highly important in the treatment of many diseases, including cancer [51].

By bridging two areas that have largely been studied independently in oncology research, this review highlights how integrating them at the drug design level may offer new directions for targeted cancer therapy. The focus on in silico methodologies underscores how computational techniques can facilitate and accelerate the identification of compounds that simultaneously regulate angiogenic and epigenetic pathways.

## 2. Methodology for Literature Search

A comprehensive review of major academic databases was conducted to identify relevant articles on computational approaches for designing multitarget inhibitors of the VEGFR and SIRT signaling pathways. Specific search terms were defined and used to search the literature: “dual VEGFR and SIRT inhibitors,” “QSAR modeling of dual VEGFR and SIRT inhibitors,” and “SBDD of dual VEGFR and SIRT inhibitors,” to ensure a thorough collection of articles addressing dual-target drug discovery strategies. The search covered the period from 2008 to 2025 and included the latest developments and methods used in the development of dual-target inhibitors. Studies were filtered and sorted by the relevance of the in silico techniques applied, including QSAR, SBDD, and molecular docking, for the identification and optimization of dual inhibitors targeting both the VEGFR and sirtuin signaling pathways. The review process included evaluating abstracts and full-text articles to ensure the inclusion of significant studies that use innovative computational methods in the development of effective anticancer drugs.

## 3. Machine Learning QSAR Modelling in VEGFR and SIRT Biochemical Space

### 3.1. Mathematical Representation of Relationships

The QSAR strategy is one of the cornerstones of in silico drug substance candidate design. These relationships enable a threefold approach to discovery: understanding the mechanism of action when only ligand information is available, predicting activity, and designing new active compounds based on mechanistic information. All three approaches are possible due to the relationship between biological activity and chemical structural properties, expressed through molecular features [52,53]. Typical mathematical forms of QSAR include multilinear regression (MLR) and partial least squares regression (PLS). In recent decades, other machine learning (ML) techniques have become crucial and rapidly developing areas in computational drug design, divided into supervised and unsupervised learning. Various ML techniques, such as support vector machines (SVM), artificial neural networks (ANN), random forest (RF), and self-organizing maps (SOM), are based on different regression and classification algorithms to represent QSARs, enabling the organization and analysis of extensive data sets [54,55]. For example, Random Forest (RF) is a machine learning approach suitable for both continuous (regression) and categorical (classification) data, depending on the desired outcomes. These cheminformatics methods help model and understand internal relationships in both small and large data sets [56].

Developed data-driven (QSAR) models are available from databases such as QSAR DataBank (QsarDB), with the aim of making in silico modelling processes and results transparent, reproducible, and accessible for further bioactivity prediction and the design of novel (multi)targeted inhibitors [57,58].

### 3.2. Three-Dimensional Geometry Representation in QSAR

Besides the QSAR approach using the 3D geometry of molecules, the popular 3D-QSAR methods in computer-aided drug design are Comparative Molecular Field Analysis (CoMFA) [59] and Comparative Molecular Similarity Indices Analysis (CoMSIA) [60]. For both, partial least squares regression analysis is used to correlate molecular descriptors with biological activity. The molecule is placed in a 3D shape with coordinates (grid). For 3D-QSAR, force field calculations are performed using the three-dimensional structures of compounds with known activity. In addition to electrostatic and steric properties, CoMSIA also considers hydrophobic properties, hydrogen bond donors, and hydrogen bond acceptors as molecular descriptors. Before calculating structural properties, ligand alignment must be performed [60]. The limitations of 3D-QSAR have been overcome by the introduction of GRid-INDependent descriptors (GRINDs) [61]. This method begins by calculating the energy of interaction between molecules and the so-called chemical probe (DRY (hydrophobic interactions), O (carbonyl oxygen, H-bond donor), N1 (H-bond acceptor), and TIP (molecule shape). The results of the 3D-QSAR model using the PLS statistical method are presented as correlograms, which present the energy of interaction between two node regions in relation to their distance. Each peak in the correlogram represents one variable or the interaction between two regions [61].

This overview is tailored in chronological order of publications and discusses chemical compound classes and the QSAR models developed for them within the VEGFR/SIRT biochemical space. This should help readers orient themselves to the groups of chemical compounds that have been shown to be important in the development of new VEGFR and SIRT inhibitors. Similarly, the following subsections provide an overview of various data-based computational models that can be used to understand molecular interactions. In the next steps, information is provided about the collected QSAR models made for VEGFR and SIRT inhibitors from different groups of derivatives and their role in designing new molecules.

### 3.3. Quantitative Structure–Activity Relationships for VEGFR Inhibitors

Sharma et al. (2007) [62] reported a QSAR study on a set of 29 derivatives of N-Phenyl-N′-{4-(4-quinolyloxy)phenyl}urea inhibiting the VEGFR-2 receptor. Multiple regression analysis (MRA) using the method of least squares was applied for model generation and predictive analysis. Molecular descriptors with negative contribution were ClogP for the whole molecule, which indicated that decreased hydrophobicity at the ortho position and less steric hindrance and para-substituent without a hydrogen-bond acceptor for the inhibition. Electronic interactions at position 3 were favourable for VEGFR-2 inhibition.

Ruan et al. (2008) [63] reported a pseudo-receptor-based QSAR study using six pseudo-probe types as molecular features on a series of 36 benzoxazole derivatives of VEGFR-2 inhibitors. The probes were designed to be virtual, aiming to develop a strategy to suggest a theory about real receptor active sites. Mutational particle swarm optimization (MPSO) was used for parameter and probe position assessment. The features employed included empty probes, electrostatic probes, steric probes, hydrophobic probes, hydrogen bond donors, and receptor probes. Optimized virtual probes provided insight into the receptor active site, emphasizing hydrogen bond and hydrophobic interactions, with partial contributions from electrostatic and steric effects.

Li et al. (2008) [64] reported MLR and nonlinear Least Squares Support Vector Machines (LS-SVMs) analyses on 32 VEGFR-2 inhibitors of pyrazine-pyridine biheteroaryl derivatives. The best model was reported with the LS-SVMs nonlinear machine learning method with five molecular descriptors derived from constitutional, topological, geometrical, electrostatic, and quantum-chemical features, with the quantum-chemical being the most influential one. The presence of a hydrogen bond plays a crucial positive contribution to ligand–receptor interactions. Heteroatom N has an important role as an electron donor, enhancing the activity of pyrazine–pyridine biheteroaryl derivatives.

Du et al. (2009) [65] conducted a 3D-QSAR study of 82 VEGFR tyrosine kinase inhibitors with activities ranging from pIC_50_ 5.8 to 9.7. They compared two alignment methods and found that docking conformer-based alignment (DCBA) was more effective. Their best CoMSIA model included steric, electrostatic, hydrophobic, and hydrogen bond donor and acceptor fields, with hydrophobic and electrostatic effects having the greatest influence. The study highlights the importance of hydrophobic and electrostatic interactions for VEGFR inhibition and the need for careful molecular targeting for reliable QSAR modelling.

Sun et al. (2010) [66] reported a simultaneously optimized genetic algorithm–support vector regression (GA-SVR) method on a series of 61 naphthalene- and indazole-based VEGFR-2 inhibitors, with better performance compared to the genetic algorithm–multilinear regression (GA-MLR) method. The best structural information indicates that a higher number of oxygen atoms is favourable for inhibitory activity. Additionally, molecular polarizability, van der Waals volume, and molecular mass play important roles. More robust substituents on aromatic rings may decrease VEGFR-2 inhibition. A bicyclic quinolyl ring moiety is favourable for VEGFR-2 inhibition.

Lan et al. (2011) [67] developed 3D-QSAR models for 32 d-angulated benzazepinone VEGFR-2 inhibitors using CoMFA and CoMSIA techniques. Compounds were aligned using the key atoms of the benzazepinone scaffold, with the most active compound selected as the template. The modelling process considered hydrophobic, electrostatic, steric, and hydrogen bonds. QSAR analysis revealed favourable structural features for activity: electron-withdrawing groups at the R1 position; bulky, electron-withdrawing and hydrogen bond-donating substituents at R2; bulky hydrogen bond-accepting substituents at R3; and small groups at R4 that may enhance potency. It was shown that the phenyl group on the benzazepinone core was necessary for the inhibitory effect, while the carbonyl group on the scaffold and the hydroxyl group on R2 played a key role in binding to residues in the ATP-binding pocket of VEGFR-2. The models were validated by cross-validation and test predictions and confirmed their reliability in identifying key molecular features that contribute to the inhibition of VEGFR-2.

Pandey et al. (2012) [68] reported QSAR modelling utilized on 32 aminopyrazolopyridine urea derivatives for VEGFR-2 inhibitors within a 2D molecular descriptors space. The model revealed four descriptors, indicating that the presence of a nitrogen atom in a certain position leads to enhanced activity, along with increasing polarizable groups in the molecules. The presence and number of aromatic rings cause enhanced hydrophobicity of the molecules not favourable for activity. Reduction of hydrogen bond donor groups in the molecules increases activity.

Zhang et al. (2012) [69] reported 3D-QSAR studies on a dataset of 53 KDR (VEGFR-2) inhibitors comprising arylphthalazines and 2-((1H-azol-1-yl)methyl)-N-arylbenzamides, with activities ranging from pIC_50_ 5.3 to 8.5. CoMFA and CoMSIA models based on hydrophobic, electrostatic, steric and hydrogen-bonding fields were developed using partial least squares (PLS) analysis. The models showed good predictive ability with cross-validated correlation coefficients for CoMFA and CoMSIA, respectively. Considering the analysis of the contour maps, it was shown that bulky groups near particular positions provided increased activity, while steric hindrances in other regions decreased activity. These results sustained valuable revelations into the structural entities for KDR inhibition.

Deeb et al. (2013) [70] reported multilinear (MLR), partial least squares (PLS) regression and non-linear principal component—artificial neural networks (PC-ANN) machine learning QSAR methods on a set of 192 VEGFR-2 inhibitors. Linear methods gave better results for predicting novel VEGFR-2 inhibitors.

Rajagopalan et al. (2013) [71] performed a Three-dimensional-QSAR study on a series of 81 novel VEGFR-2 inhibitors belonging to 4-aminopyrimidine-5-carbaldehyde oxime and N-phenyl-N′-{4-(4-quinolyloxy)phenyl}urea derivatives. Using pharmacophore-based alignment and atom-based 3D-QSAR modelling in PHASE [72], the compounds were classified as active and inactive based on their pIC_50_ values. The best pharmacophore model included features such as hydrogen bond donors and acceptors, and aromatic rings critical for activity. Partial least squares regression was used to correlate the molecular features with biological activity. 3D contour maps identified favourable regions for hydrophobic interactions, hydrogen bond donors and electron-withdrawing groups that enhance inhibition of VEGFR-2. The model highlighted the importance of urea group substitutions and phenyl ring positions in modulating activity and provided useful insights for the further design of potent inhibitors.

Nekoei et al. (2015) [73] reported machine learning QSAR based on genetic algorithm (GA) multiple linear regression (GA-MLR) and support vector machine (GA-SVM) to perform modelling on a 32 4-aminopyrimidine-5-carbaldehyde oxime derivatives as selective VEGFR-2 inhibitors, divided randomly into training and test set. The best performance with the same molecular descriptors was achieved using GA-SVM analysis.

Balupuri et al. (2015) [74] reported a 3D-QSAR study on a series of tetrahydro-3H-imidazo[4,5-c]pyridine derivatives as VEGFR-2 kinase inhibitors. Modelling was performed with the dataset of 36 compounds with IC_50_ inhibitory activity converted to pIC_50_ values. The lowest energy conformation of the most active compound was used as a template for alignment of the optimized molecular structures. Based on the evaluation of different partial atomic charge schemes, it was found that Gasteiger-Marsili charges provided the most statistically significant models. 3D-QSAR CoMFA and CoMSIA were used to investigate steric, electrostatic, hydrophobic and hydrogen bonding donor and acceptor fields. The CoMFA model showed strong internal with moderate external validation parameters. Contour maps emphasized the importance of steric bulk near the B-ring and halogen substitutions at the 6-position of the A-ring for enhanced inhibitory activity. The study provided valuable insights into structural features critical for the inhibition of VEGFR-2 kinase and supported further rational design of potent inhibitors.

Sefiddashti et al. (2021) [75] performed QSAR modelling using artificial neural networks and multiple linear regression with five molecular descriptors on 33 furo[2,3-d]pyrimidine and thieno[2,3-d]pyrimidine VEGFR-2 inhibitors carrying biarylamide or biarylurea across an NH or ether linker. The 2D autocorrelation group of molecular descriptors—RDF035u, Mor24v, EEig11r, ATS3v, and G2s—were identified as the most influential. Among these, ATS3v contributed negatively to activity. The artificial neural network provided better results for predicting similar compounds.

Tong et al. (2021) [76] reported 2D hologram QSAR (HQSAR) and topomer 3D-COMFA analysis using 44 derivatives of 6-amide-2-aryl benzoxazole/benzimidazole derivatives, inhibiting VEGFR-2 receptor. HQSAR considers molecular features described as HL (hologram length), FD (fragment discrimination) and FS (fragment size). The most promising HQSAR model revealed the best features within A/B/C/CH for fragment differentiation, 7–10 for fragment size and 97 for hologram length. Structural modification of R-based structures of lead molecules from the dataset was assessed within joint contribution on 2D-QSAR maps and 3D-QSAR contour maps.

El-Meguid et al. (2022) [77] reported a machine learning multilinear regression QSAR model on a series of 22 benzothiazole derivatives inhibiting the VEGFR-2 receptor. The 2D molecular descriptors SMR_VSA4, SMR_VSA5 and the 3D dipoleZ descriptor were selected for gaining better insight into structural space arrangement for enhanced inhibitory activity. Based on the analysis of the molecular features, it was shown that the kinase inhibition potency is related to voluminousness and molecular partial charge distribution. Results indicated that structural modifications are correlated with the size of a molecule and intramolecular electrostatic interactions.

Abdullahi et al. (2023) [78] subjected 33 quinoxaline derivatives with VEGFR-2 inhibitory activity to multilinear regression analysis using a genetic function algorithm for 2D molecular descriptor selection. Molecular descriptors SpMax8_Bhs, GATS5e, and GATS3i exhibit positive influence, while GATS8i and VR2_Dt descriptors exhibit negative influence on activity. Five novel compounds were designed based on structural information from molecular descriptors and additional structure-based guided analysis.

Banerjee et al. (2023) [79] reported a QSAR analysis of 98 thiourea-based VEGFR-2 inhibitors divided into five clusters with the k-means cluster analysis (k-MCA) method. Diverse molecular descriptors, including 2D and fingerprints, were used in correlation with VEGFR-2 pIC_50_ activity. A genetic functional algorithm with partial least squares regression (GFA-PLS) and hologram PLS-based QSAR (PLS-HQSAR) study for fingerprints was employed for modelling. Classification methods were used on a particular molecular fragment of thiourea derivatives as a potential structural alert responsible for activity. These alerts are curated using the Python-based SARpy tool for structure–activity in the form of SMILES format. A Bayesian classification study was also performed to predict probabilities within the dataset. Molecular descriptors SpMax8_Bhs, GATS5e, and GATS3i exhibit a positive influence on the inhibitory activity of the quinoxaline molecules, while GATS8i and VR2_Dt descriptors exhibit a negative influence. The studies revealed the importance of the amine-substituted quinazoline phenyl ring fragment and a higher number of heteroatoms, like nitrogen, responsible for high inhibitory activity and VEGFR-2 binding. The presence of methoxy groups has a decreasing activity trend, while thiourea, the urea moiety and hydrazine were favorable for the inhibition and binding affinity to the protein.

Tripathi et al. (2023) [80] performed machine learning based modelling of VEGFR-2 inhibitors using the KNIME platform to calculate molecular and fingerprint-based descriptors and machine learning classification algorithms in five different machine learning algorithms, including linear regression (LR), decision tree (DT), random forest (RF), k-nearest neighbor (kNN), and gradient boosted tree (GBT). The dataset was formed by obtaining structurally diverse 5120 molecules from the BindingDB database, separated into training, test, and validation sets, with the ratio of 70:15:15, respectively, represented as active and inactive compounds. The best performance was achieved with the gradient boosted tree algorithm.

Baammi et al. (2023) [81] compiled a dataset of 23 triazolopyrazine derivatives tested against the breast cancer cell line MCF-7. Using nineteen compounds as a training set and four for external validation, 3D-QSAR models were developed using CoMFA and CoMSIA techniques. The CoMFA model considered steric and electrostatic fields, while CoMSIA considered hydrophobic, hydrogen bond donor and acceptor groups together with steric and electrostatic factors. Partial least squares (PLS) regression with leave-one-out cross-validation yielded statistically robust models with strong predictive power. Analysis of steric CoMFA contour maps showed that bulky substituents near certain positions of the triazolopyrazine core enhanced inhibitory activity, suggesting favourable steric interactions in the receptor binding pocket. Electrostatic contour maps showed that regions of negative charge were favoured in certain molecular regions, suggesting strong electrostatic complementarity with VEGFR-2. Analogous to that, the hydrophobic fields of CoMSIA highlighted the weightiness of hydrophobic groups in increasing the activity, which is probably related to improving ligand–receptor binding affinity through non-polar interactions. The fields of the hydrogen bond donors and acceptors indicate that properly oriented groups that can form hydrogen bonds, contribute considerably to improved inhibitory activity. These combined steric, electrostatic, hydrophobic and hydrogen bonding interactions described by the 3D-QSAR models provided valuable insights into the molecular features critical for inhibition of VEGFR-2 in breast cancer cells.

Verma et al. (2024) [82] reported a QSAR model on structurally diverse known VEGFR-2 inhibitors with forward stepwise multilinear regression to predict the inhibitory activity of natural compounds. The QSAR model revealed five 2D molecular features favourable for inhibitory activity prediction including the electro-topological state of potential hydrogen bonds, atom types, maximum topological distance matrix and 2D autocorrelation descriptor. Natural compound lavendustin A, 3′-O-acetylhamaudol, and arctigenin were considered as future potential in VEGFR-2 kinase inhibition.

Gupta et al. (2024) [83] reported eight QSAR model using 118 benzo-fused heteronuclear derivatives to enhanced potency of VEGFR-2 inhibitors generated within regression Monte Carlo algorithm. A 2D representation was used to assess the combination of SMILES-based with different graph-based optimal descriptors. Derived molecular features were used for existing structural modification to design novel inhibitors together with molecular docking and molecular dynamic simulation. Seven novel compounds named YS01-YS07 are predicted to have enhanced pIC_50_ values, together with better docking results for ligand-protein interactions.

Abdullahi et al. (2025) [84] reported a multilinear regression QSAR model on a series of 45 benzoxazole/benzimidazole derivatives for VEGFR-2 using a genetic function algorithm (GFA) for molecular descriptor selection. The most important descriptors were 2D SpMax5_Bhp, ATS5v, and AATSC7v, and MATS5c descriptors. New compounds were designed based on structural modifications involving electron-rich substituents (-OH, -NH_2_, F). Additional structure-based methods were used to verify potential of the molecules.

### 3.4. QSAR Models for SIRT Biochemical Structural Space

Park et al. (2009) [85] reported 3D-QSAR COMFA and COMSIA on a series of 33 imidazothiazole and oxazolopyridine derivatives inhibiting SIRT1 and revealing better COMSIA performance. Electrostatic and steric features analysis of the model show that redesign of existing imidazothiazole and oxazopyridine derivatives, requires structural modification on the aryl group directly attached to the central amide -NH.

Alvala et al. (2012) [86] reported 3D-QSAR studiy on a series of acridinediones inhibiting SIRT1 protein. Analysis revealed the importance of hydrophobic and non-polar moieties substitution at the ortho position rather than para or meta substitution, as essential for bioactivity based on the most active SIRT1 inhibitor with IC_50_ = 10.13 ± 0.08.

Kokkonen et al. (2014) [87] reported 3D-QSAR COMFA analysis on a series of 79 substrate-based SIRT1 inhibitors containing different favourable moieties replacing acetyl lysine. The predictiveness of the QSAR model was demonstrated with statistical parameters for the training and test set together with the corresponding COMFA contour maps in designing 13 pseudopeptidic SIRT1 inhibitors.

Chuang et al. (2015) [88] reported 3D-QSAR COMFA modelling on a series of 46 2-anilinobenzamide-related SIRT2 inhibitors. The steric and electrostatic contour maps from the 3D-QSAR analysis supported by MD and docking result revealed that structural modification at the meta position of the aniline moiety can form π staking with the F119 aromatic residue, while modification in para position is not favorable. The SIRT2 binding pocket is mostly hydrophobic with dominating aromatic and aliphatic amino acids. The results suggested that structural modifications of the best candidate for SIRT2 inhibitor should exclude hydrogen bonding groups directed towards SIRT1 R207 and SIRT3 Q109.

Pulla et al. (2016) [89] reported-energy based pharmacophore modelling and 3D-QSAR on a series of 79 SIRT1 inhibitors consisted of indole, aurones, thioacetyl lysine, pyrimidine carboxamide, and sirtinol derivatives. The 3D-QSAR model was used to generate contour maps, for analyzing the importance of certain functional groups at specific positions toward biological activity. The model was developed with PLS statistical method, and it was used in a virtual screening protocol to search for the most active SIRT1 inhibitors using various databases including 3D Asinex and in-house databases, revealing a compound with IC_50_ = 4.3 μM, as the best SIRT1 inhibitor.

Kumar and Chauhan (2017) [90] reported a QSAR model for SIRT1 inhibitory activity employing linear regression on a series of 45 compounds with SMILES based descriptors within the Monte Carlo optimization method using Coral software. Good statistical parameters revealed robustness and predictability. Molecular features and fragments analysis demonstrated that the presence of branching (‘(…………’), the presence of double bond with oxygen and branching (‘O… = ……’, ‘= …(………’) and the presence of nitrogen with branching (‘N…(………)’) were favourable for SIRT1 activity increase, while the presence of oxygen with branching (‘O…(………’) gave negative contribution for the SIRT1 activity. Study on a larger dataset for both SIRT1 and SIRT2 inhibitors with Monte Carlo method and SMILES representation was performed by Worachartcheewan et al. [91].

Khanfar et al. (2017) [92] reported multilinear regression (MLR) and k-nearest neighbor (kNN) developed based on genetic function algorithm (GFA) for pharmacophores and molecular descriptor selection, SIRT2 inhibitors bioactivity prediction. Ligand efficacy was used as dependent variable in 96 SIRT2 inhibitors. The kNN non-linear approach was used to overcome collinearity and descriptor non-linearity related to prediction in linear regression QSAR model. kNN-based analysis revealed different pharmacophores comparing to the MLR model with different sets of favourable features, demonstrating the importance of different QSAR modelling approaches to estimate binding phenomena within large binding pockets like in the SIRT2 protein.

Kumar and Chauhan (2017) [93] reported a machine learning classification model on 67 activators and 45 inhibitors for SIRT1 modulators using the Monte Carlo optimization method and SMILES based descriptors. Molecular features and fragments revealed that the presence of nitrogen and hydrogen (‘n…H……’) and presence of double bond with an oxygen atom (‘O…=………’) have positive contribution for the SIRT1 inhibitory activity.

Gupta and Choi (2018) [94] reported an integrated 2D and 3D-QSAR approach on a series of imidazothiazole and oxazolopyridine derivatives as SIRT1 activators. Steric, electrostatic, hydrophobic and hydrogen bond acceptor interactions revealed to be favourable for human SIRT1 activation which was in consensus with 2D methods.

Agrawal et al. (2018) [95] reported pharmacophore and atom-based 3D-QSAR study on a series of thieno[3,2-d]pyrimidine-6-carboxamide derivatives. Significant statistical internal and external parameters revealed predictive QSAR model developed based on PLS regression analysis. The analysis of pharmacophore model revealed the importance of crucial features for SIRT2 enzyme in binding affinity of two hydrogen bond acceptors, two hydrogen bond donors and one hydrophobic feature.

Chauhan and Kumar (2018) [96] reported a hierarchic 3D-QSAR method (HiT QSAR) on a series of 65 SIRT1 activators with a pEC_1.5_ endpoint (the concentration of an activator required to produce a 1.5-fold activation of the enzyme) using simplex representation of molecular structure employing PLS statistical analysis. Model results were obtained as a consensus model exhibiting following features for enhanced activity: atomic charges (42%), electronegativity (21%), H-bonds donor/acceptor (1%) and lipophilicity (3%). The van der Waals attraction and Sybyl type revealed negative influence.

Pratiwi et al. (2019) [97] reported a QSAR model analysis developed with the MLR statistical method on a series of chemically diverse imidazothiazole, oxazolopyridine, and azabenzimid scaffolds divided into three sets to achieve better insight into structure–activity relationship. All data were taken from the ChEMBL database. QSAR model results served as a basis for designing 181 structurally similar SIRT1 activators tested with the model’s equation. The QSAR model of imidazothiazole analogues revealed that electronegativity, charge, and polarizability influenced bioactivity the most. The QSAR analysis of oxazolopyridine derivatives showed that electronegativity was the most influential for bioactivity. The QSAR result of azabenzimidazole compounds revealed the importance of mass, atomic polarizabilities and the WHIM index. Several newly designed SIRT1 activators were employed for external validation of the model.

Ferreira et al. (2019) [98] reported a diverse QSAR modelling approach on a series of 75 3′-phenethyloxy-2-anilinobenzamide derivatives as SIRT2 inhibitors using the PLS statistical approach. Models were reported using 2DHQSAR employing molecular fingerprints of fragments (holograms) and a 3D structure computational approach with 3D-COMFA and 3D-COMSIA. The predictive capabilities of the QSAR models were demonstrated with the statistical parameters. Molecular feature analysis revealed insight into potential structure modification to gain enhanced SIRT2 inhibitors. The analysis revealed the importance of the 2-anilinobenzamide group and the introduction of a third ring with favourable polar interactions with halogens F, Cl and Br in the meta position. Additionally, the analysis emphasized the significance of two distinct hydrophilic and hydrophobic regions that could be extended with larger hydrophobic groups.

Worachartcheewan et al. (2021) [91] reported machine learning regression QSAR models using the Monte Carlo method for SIRT1 and SIRT2 inhibitors using Optimal descriptors (DCW) from 2D structures represented as SMILES using Coral software, in total for SIRT1 310 and SIRT2 345 compounds. Increase in inhibitory activity for SIRT1 was associated with molecular features such as the presence of a double bond with branching (‘C…(…=…’, ‘C…=…(…’), a nitrogen atom with branching (‘N………’, ‘N…(……’), a double bond with an oxygen atom (‘=…O…(…’, ‘O…=……’) and cycles with branching (‘1………’, ‘C…1……’, ‘1…C…(…’). Decrease in inhibitory activity for SIRT1 was described within the molecular features described as the presence of a double bond (‘=………’, ‘C…=……’), the presence of branching (‘(…C…(…’) and the presence of double bond and cycles (‘=…1……’). Results from QSAR study for SIRT2 biochemical space revealed the importance for increasing activity within molecular features like branching (‘(…C…(…’, ‘(…(……’), the presence of a double bond with an oxygen atom and branching (O’…=……’, ‘O…=…(…’, ‘O…(…C…’), the presence of cyclic (‘1………’, ‘C…1……’) and the presence of a nitrogen atom with branching (‘N…(……’). Decreasing activity for SIRT2 was demonstrated with the presence of a double bond with branching (‘=…(……’, ‘C…(…=…’, ‘=…O…(…’, ‘[…(…=…’), the presence of a double bond with cyclic (‘=…C…1…’) and the presence of cyclic with branching (‘1…C…(…’, ‘[…1……’, ‘H…[…1…’).

Bharadwaj et al. (2021) [99] reported and Atom and Field-based 3D-QSAR modeling on a series of 39 known selective SIRT2 inhibitors, including SirReal2 inhibitors. The model was used for drug repurposing analysis to predict the activity of selected FDA-approved drugs as possible SIRT2 inhibitors using the PLS regression method. Nintedanib drug exhibited predicted pIC_50_IC50 = 6.051 µM (Atom-based QSAR) and pIC_50_ = 5.94 µM (Field-based QSAR), was the most promising repurposing drug for SIRT2 inhibition.

Kessler et al. (2021) [100] reported a machine learning QSAR model for the binary classification on a chemically diverse dataset consisting of 234 SIRT2 inhibitors from the ZINC15 database. Additionally, results were further applied for machine learning virtual screening analysis using an FDA subset (107 compounds). To conduct structure-based virtual screening binding analysis to deepen the process of discovery of new SIRT2 inhibitors, AutoDock Vina was employed. In total, 43 compounds revealed the best inhibitory performance. The article demonstrated the joint effort of ligand and structure-based analysis in possible drug repurposing or insight into structure re-designing using molecular features.

Sahar Ilaghi-Hoseini and Zahra Garkani-Nejad (2022) [101] reported a multilinear regression QSAR and a nonlinear regression support vector machine (SVR) model within the same four molecular descriptors information on 33 compounds of 2-((4,6 dimethyl pyrimidine-2-yle) thio)-N-phenyl acetamide derivatives as SIRT2 inhibitors. Molecular descriptors were related to Burden eigenvalue descriptors (BELV2), two 2D autocorrelation descriptors (GATS6e, GATS8p) and the RDF (radial distribution function) 3D descriptor.

Lim et al. (2022) [102] reported an approach combining QSAR modeling and DNA Encoded Library (DEL) dataset within a regression method that avoids the binarization and aggregation of enrichment data. This approach was used on a DEL dataset of 108,528 compounds screened against carbonic anhydrase (CAIX), and a dataset of 5,655,000 compounds screened against soluble epoxide hydrolase (sEH) and SIRT2 inhibitors employing an uncertainty-aware probabilistic loss function.

Djokovic et al. (2023) [103] reported a regression and classification machine learning-based SIRT2i_Predictor for inhibitory and selectivity prediction. Five machine learning algorithms with different molecular feature selection, including fingerprints within the dataset of 1797 inhibitors from the ChEMBL database, were employed. Datasets of SIRT inhibitors with corresponding pIC_50_ and Inh% were employed for model development, divided into four datasets. Regression-based QSAR analysis was used to model 1002 SIRT2 inhibitors with pIC_50_ as the dependent variable. Additionally, datasets from 2 to 4 were used for classification analysis, including SIRT1-3 isoforms and pIC_50_ and Inh% for biological activity. The datasets covered diverse chemical space and also different bioassay protocols. In conclusion, the SIRT1/2 model showed excellent predictive power, whereas the SIRT2/3 models demonstrated lower quality on topologically dissimilar compounds. The applicability of the SIRT2i predictor was tested in a novel structure-based virtual screening analysis, including 200,000 compounds from the SPECS database, revealing its application for virtual screening (VS), lead optimization, experimental evaluation, repurposing studies, or more complex precision-medicine pipelines.

Ilic et al. (2024) [104] reported an integrated in silico approach with 3D-QSAR and machine learning techniques on a series of 86 nicotinamide-based SIRT2 inhibitors using *Ki values*. The 3D-QSAR model was developed using alignment-independent GRIND molecular descriptor pharmacophores employing regression PLS analysis, obtaining significant model parameters. A machine learning QSAR model was performed based on molecular docking results for the SIRT1–3 isoforms, within classification analysis. SIRT1/2 model using the Naive Bayes algorithm and SIRT2/3 using the k-nearest neighbors algorithm models were developed to gain the selectivity of inhibitors for SIRT1/2 and SIRT2/3. SAR analysis revealed the importance of specific structural motifs, including steric features, a secondary amide moiety as a hydrogen bond donor and a nitrogen moiety within the pyridine ring as a hydrogen bond. Based on pharmacophore analysis from GRINDs derived from molecular interaction fields and classification model selectivity, new nicotinamide-based inhibitors were designed and predicted with the 3D-QSAR model.

Alkhatabi et al. (2024) [105] reported a study investigating the influence of converting cyclic inhibitor peptide (S2iL5) of SIRT2 into a non-cyclic form. To additional analysis, QSAR modelling of peptide inhibitors for SIRT2 to predict protein–ligand interactions was reported. In total, 876 protein–peptide complexes from the PDB database were used for machine learning QSAR model development. Machine learning algorithms, like Random Forest, Ridge Regression, Gradient Boosting, and XGBoost, were employed for model development. The XGBoost algorithm was used for virtual screening of a peptide library, revealing three in silico peptides after clustering analysis.

Luo et al. (2025) [106] reported a 3D-QSAR COMFA and COMSIA model developed on 64 reported 5-((3-amidobenzyl)oxy) nicotinamide compounds. Modelling was performed using PLS analysis. COMFA and COMSIA contour maps analysis revealed that adding negatively charged groups with hydrophobic properties to the imidazole ring within the molecule could enhance the inhibitory activity of SIRT1 inhibitors.

## 4. SBDD of Multi-Target Ligands for VEGFR, SIRT and Other Cancer-Related Targets

### 4.1. Structure-Based Drug Design (SBDD)

Structure-based drug design (SBDD) is a field in computational chemistry that serves as an indispensable starting point for discovering and optimizing lead molecules, based on knowledge of the crystal structure of the target [107]. The need for this approach is particularly evident in developing therapies for complex challenges, such as multisystem diseases, where it is necessary to target multiple proteins simultaneously while maintaining both affinity and selectivity [108]. To design dual inhibitors using this method, the structural features and binding sites of both targets must first be determined. The subsequent design of new ligands may involve creating entirely new molecular scaffolds or modifying existing structures to improve current inhibitors. Additionally, large compound databases can be filtered using various virtual screening methods [109]. In later stages of discovery, it is important to predict the binding mode of the designed ligands and their potential interactions with amino acid residues in the target’s active site, which is achieved through computational molecular docking techniques [110]. This is followed by experimental in vitro validation, in which enzymatic and cellular assays are performed on compounds previously recognized as the best candidates.

### 4.2. Dual VEGFR/HDAC Inhibitors

Dual VEGFR/HDAC inhibitors are an emerging class of compounds in cancer research that hold significant promise for improving cancer therapy, particularly due to their potential synergistic effects on both tumor growth and the tumor microenvironment. The main importance of developing dual VEGFR/HDAC inhibitors is to target two distinct but interconnected pathways in tumor biology: angiogenesis (through VEGFR signaling) and gene silencing (through HDAC activity). VEGFR inhibitors are pharmacologically active compounds that block the VEGFR, preventing the formation of new blood vessels and thus depriving tumors of the resources necessary for their development [111]. HDAC inhibitors, on the other hand, block the HDAC enzyme, leading to increased histone acetylation and activation of tumor suppressor genes, which can induce apoptosis and differentiation of cancer cells [112]. HDAC inhibitors have shown promise in various cancers, including hematological malignancies and solid tumors [113]. In addition to simultaneously targeting both VEGFR and HDAC within a complex network of signaling pathways, dual VEGFR/HDAC inhibition may reduce the likelihood of resistance development, a common problem in clinical practice [114]. In this context, HDAC inhibitors can re-sensitize tumors to therapies to which they have become resistant, such as anti-angiogenesis drugs. Simultaneous targeting of both VEGFR and HDAC could expand the range of applications, given that HDAC inhibitors also have effects on neurological disorders and VEGFR inhibitors on complications of diabetes. The protein structures of the active sites of VEGFR and HDAC do not exhibit a high degree of similarity, which is consistent with their different biological activities. However, if dual inhibitors contribute to a measurable cellular effect, one could speak of the existence of functional similarity [115].

**Figure 2:**
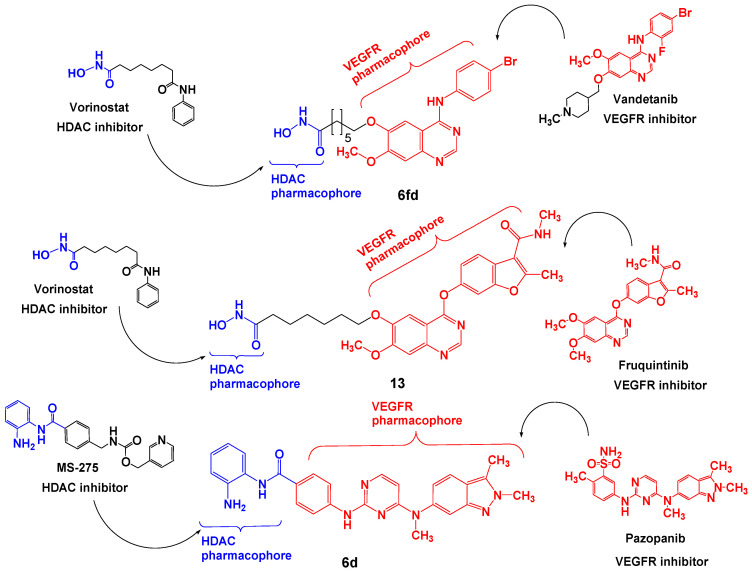
Chemical structures of representative dual VEGFR/HDAC inhibitors and single-acting agents used for their design. VEGFR pharmacophores are coloured red and HDAC pharmacophores are coloured blue [116,117,118].

Aiming to improve treatment outcomes by targeting multiple pathways that contribute to cancer pathology, SBDD has been successfully implemented in the design of dual VEGFR/HDAC inhibitors [116,117,118]. It is well established that HDAC inhibitors comprise three pharmacophores in their structure, which served as a starting point for rational design [119]. Specifically, on one side of the molecule is a functional group that chelates zinc (ZBG), the other side has a functional group with affinity for binding to amino acid residues on the outer surface of the enzyme (CAP), and a hydrocarbon group acts as a linker, forming a bridge between these two groups [118]. Zang et al. designed dual VEGFR/HDAC inhibitors based on pazopanib to simultaneously target cancer epigenetics and angiogenesis [118]. Hydroxamic acid and ortho-aminoanilide were used as zinc-chelating groups, which were connected via a linker to the phenyl group of pazopanib disclosed to the solvent, thus defining the basic structure of a new series of dual inhibitors. Sybyl X_2.1 software (Tripos International, St. Louis, MO, USA) was applied for molecular docking on the crystal structures of VEGFR-2 (PDB code 3CJG) and HDAC2 (PDB code 5IWG) to analyze the binding modes of the compounds designated as representative by the authors, **6d** (Figure 2) and **13f**. Interactions in a comparable binding mode of **6d** and **13f** in relation to pazopanib were observed in the ATP binding pocket of VEGFR-2, as well as overlapping of **6d** and MS-275 in the active site of HDAC2 and **13f** and SAHA in HDAC6.

A series of 4-(benzofuran-6-yloxy)quinazoline-based derivatives was created using pharmacophore fusion methodology to connect the structure of fruquintinib with the pharmacophores of HDAC inhibitors [116]. Biological activity evaluations indicated a strong inhibitory effect against VEGFR-2 (IC50 = 57.83 nM) and HDAC1 (IC50 = 9.82 nM), suggesting that compound **13** (Figure 2) could be a potent dual VEGFR-2/HDAC inhibitor. Autodock Vina was applied for molecular docking of compound **13** with the VEGFR-2/naphthamide inhibitor complex (PDB: 3B8R) and the HDAC1/SAHA complex (PDB ID: 1C3S). Results showed that compound **13** exhibited a binding mode comparable to fruquintinib at the ATP binding pocket of VEGFR-2. Additionally, important interactions, including chelation with Zn^2+^, hydrogen bonds, and hydrophobic interactions, were observed in the active site of HDAC1, providing significant evidence for the potential dual activity of compound 13 against VEGFR-2 and HDAC1.

**Figure 3:**
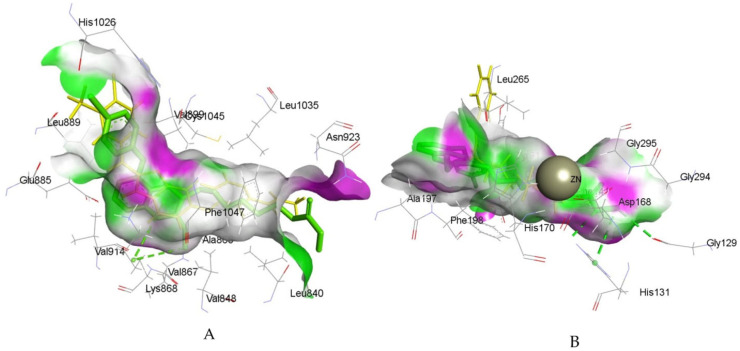
Visualization of the binding mode of compound 6fd (dual VEGFR-2/HDAC inhibitor) in the active sites of (**A**) VEGFR-2 (PDB: 2QU5) and (**B**) HDAC (PDB: 1C3S). Compound **6fd** is represented with green sticks, while the reference ligands are represented with yellow sticks.

By connecting the N-phenylquinazolin-4-amine moiety, a pharmacophore of the VEGFR-2 inhibitor vandetanib, and the hydroxamic acid side as a ZBG of the HDAC inhibitor vorinostat, Peng et al. created a series of dual VEGFR-2/HDAC inhibitors [117]. Biological evaluation indicated a strong inhibitory effect of compound **6fd** (Figure 2) against HDAC (IC_50_ = 2.2 nM) and VEGFR-2 (IC_50_ = 74 nM). Additionally, a molecular docking study, using the Discovery Studio 3.1/CDOCKER protocol, illustrated a common mode of interaction for compound **6fd** at the ATP-binding cavity of VEGFR-2 (PDB: 2QU5) and the active site of Histone Deacetylase-Like Protein (HDLP) (PDB: 1C3S). Results showed that compound **6fd** establishes hydrogen bonds with Asn923 and Leu840, as well as π-cation interactions with Lys868, as important interactions in the VEGFR-2 binding site. Hydrogen bonds with Tyr297, His131, and Gly129 were observed as important interactions in the binding mode with HDLP, indicating that **6fd** could be a favorable lead for further optimization in cancer therapy investigation. The docked binding mode of **6fd** overlaid with reference ligands in the active site of VEGFR-2 and HDAC, obtained using GOLD 2022.3.0 Software [120] and visualized in Discovery Studio software v24.1.0.23298 [121], is shown in Figure 3.

### 4.3. Dual VEGFR/EGFR Inhibitors

Dual EGFR (epidermal growth factor receptor) and VEGFR-2 inhibitors are increasingly important in successful cancer therapy because they influence processes involved in tumorigenesis, including proliferation and vascularization. EGFR is a receptor found on the surface of various cancer cells, and its activity can be increased in many ways [122]. Since activation of these receptors initiates cellular activities that promote cell division and survival, their inhibition could reduce tumor growth and induce apoptosis [123]. Dual EGFR/VEGFR-2 inhibitors achieve their effects by acting on cell proliferation, for which EGFR is responsible, and on angiogenesis, which is controlled by VEGFR. The development of new VEGFR-2/EGFR inhibitors is a growing need that could contribute to greater therapeutic success and help overcome potential resistance to currently available therapies [123]. It is also important to consider the similarities in the binding positions of VEGFR and EGFR proteins, especially since both are tyrosine kinases that participate in signal transmission [124]. On the other hand, their ligand-binding regions differ in sequence, structure, and function [124]. VEGFR and EGFR have similar outer membrane proteins containing many cysteine residues important for ligand interaction, but their active sites differ because their natural ligands have different chemical structures [125].

**Figure 4:**
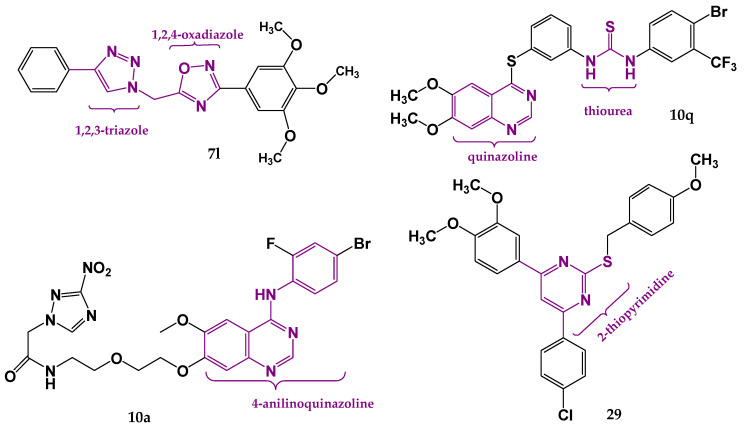
Chemical structures of representative dual VEGFR/EGFR inhibitors. Scaffolds as a core for the design of new derivatives are colored purple [126,127,128,129].

A series of quinazoline- and thiourea-containing analogues was created regarding sorafenib structure modifications to develop dual VEGFR/EGFR inhibitors [126]. Biological evaluation identified compounds **10b** (IC_50_ (VEGFR) = 0.05 μM and IC_50_ (EGFR) = 0.02 μM) and **10q** (IC_50_ (VEGFR-2) = 0.08 μM and IC_50_ (EGFR) = 0.01 μM) as having the most pronounced inhibitory activities. For compound **10q** (Figure 4), molecular docking was carried out at the active sites of EGFR (PDB: 2ITY) and VEGFR-2 (PDB: 4ASD) using Tripos Sybyl-x2.0 software (2.0, Tripos Inc., St. Louis, MO, USA). Results showed important interactions, including hydrogen bonds with Pro794, Met793, and Lys745, and hydrophobic interactions with Phe795, Met793, and Leu718 amino acid residues in the active site of EGFR, indicating that **10q** could bind well at the considered binding sites. Critical interactions observed at the ATP-binding cavity of VEGFR-2 kinase include hydrogen bonds with Asp800 and Lys745, and hydrophobic interactions with Phe795, Leu844, Met793, Val726 and Leu718 amino acid residues.

To increase the specificity of tyrosine kinase inhibitors for tumor cells, Wei et al. designed 4-anilinoquinazoline analogues as hypoxia-selective dual VEGFR-2/EGFR inhibitors [127]. The chemical structure of vandetanib, an inhibitor of VEGFR and EGFR, was modified at the aniline moiety, and a 3-nitro-1,2,4-triazole group was introduced to the side chain. Further in vitro assessment indicated that compounds **10a** (IC_50_(VEGFR-2) = 36.78 nmol/L and IC_50_(EGFR) = 5.90 nmol/L) and **10e** (IC_50_(VEGFR-2) = 1196.59 nmol/L and IC_50_(EGFR) = 3.11 nmol/L) were the most effective and safest. For compound **10a** (Figure 4), a molecular docking study was conducted by the Glide Dock method with Schrödinger Release 2019-2 (Schrödinger, LLC, New York, NY, USA) with the crystal structures of VEGFR-2 (PDB: 2RL5) and EGFR (PDB: 4I23), confirming that **10a** may retain a binding mode related to vandetanib. Significant interactions at the EGFR binding site include a hydrogen bond between the quinazoline moiety of **10a** and Met793, with no additional hydrogen bond between the 3-nitro-1,2,4-triazole side chain and EGFR. In the active site of VEGFR-2, an important hydrogen bond is observed between the quinazoline of **10a** and Cys919 of VEGFR-2, as well as additional hydrogen bonds with Thr916 and Asn923 amino acid residues in the VEGFR-2 binding site. The in vitro assays and molecular docking studies provide a basis for further optimization of lead compounds in targeting hypoxic tumors.

Comprising a 1,2,4-oxadiazole core found in known anticancer agents and a 1,2,3-triazole-scaffold with inhibitory potency against VEGFR-2, Mahmoud et al. reported 1,2,3-triazole/1,2,4-oxadiazole hybrids as dual EGFR/VEGFR-2 inhibitors [128]. A representative structure of compound **7l** is shown in Figure 4. The strongest antiproliferative activitiy was observed for **7j** (IC_50_ (VEGFR) = 4.70 ± 0.04 nM and IC_50_(EGFR) = 89 ± 08 nM), 7k (IC_50_ (VEGFR) = 3.80 ± 0.03 nM and IC_50_(EGFR) = 82 ± 07 nM), and **7l** (IC_50_ (VEGFR) = 2.40 ± 0.02 nM and IC_50_(EGFR) = 76 ± 06 nM). Additional docking simulations performed using Molecular Operating Environment (MOE 2019.0102, 2020; Chemical Computing Group, Canada) with crystal structures of VEGFR-2 (PDB: 4ASD) and EGFR (PDB: 1M17), provided insight into structural features important for fitting in the binding pockets. Important hydrogen bond interactions in the EGFR binding site were observed for **7i** (Leu764) and **7l** (Lys721). Alternatively, binding modes were settled by π–H interactions with Cys773 and Gly772 at the entrance of the binding place, showing that a better interaction pattern can be achieved with the m-halophenyl rather than the p-halophenyl moiety. The best binding modes were noticed for **7l** and **7k,** which form pi–H interaction with Leu840. Since hydrogen bond formation was lacking, it was concluded that introducing a hydrogen bond-forming substituent at the phenyl ring is required for optimal fitting.

**Figure 5:**
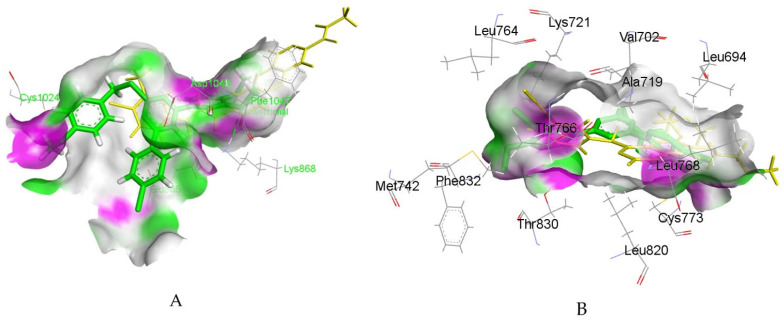
Visualization of the binding mode of compound **29** (dual VEGFR-2/EGFR inhibitor) in the active sites of (**A**) VEGFR-2 (PDB: 3WZE) and (**B**) EGFR (PDB: 1M17). Compound **29** is represented with green sticks, while reference ligands are represented with yellow sticks.

Combining the aryl ring with 2-thiopyrimidines, Mostafa et al. designed a series of 4,6-diaryl pyrimidines [129]. Their starting point was the idea that pyrimidine derivatives can form hydrogen bonds with proteins and nucleic acids, so modifications of the ring may affect their antiproliferative effects. The most pronounced antiproliferative activities were observed for **22** (IC_50_ (VEGFR) = 1.15 ± 0.01 nM and IC_50_(EGFR) = 74 ± 5 nM) and **29** (IC_50_ (VEGFR) = 1.60 ± 0.01 nM and IC_50_(EGFR) = 72 ± 5 nM). Molecular docking procedures were performed using Molecular Operating Environment (MOE^®^), version 2009.10 (Chemical Computing Group ULC, Montreal, QC, Canada). Docking results indicated binding modes of the most promising derivatives to the binding sites of EGFR (PDB: 1M17) and VEGFR-2 (PDB: 3WZE). Both compounds formed important interactions with the VEGFR-2 binding site, including hydrogen bonds with essential amino acid residues Glu885 and Lys868, stabilizing the VEGFR-2 active site. In the active site of EGFR, compound **29** (4-chloro derivative) formed a hydrogen bond with Arg817, while in the case of compound **22** (methoxy-substituted derivative), two hydrogen bond formations were observed, with Arg817 and Lys721. Therefore, it was concluded that derivatives possessing the methoxy group expressed more convenient modes of attachment than other derivatives. The required interactions of **29** in accordance with reference ligands in the active sites of VEGFR-2 and EGFR, obtained using GOLD 2022.3.0 Software [120] and visualized in Discovery Studio software v24.1.0.23298 [121], are shown in Figure 5.

### 4.4. Dual Inhibitors of SIRT and Other Cancer-Related Targets

Aiming to provide effective, safe, and more sustainable cancer therapies, the idea of designing dual inhibitors targeting sirtuins and other cancer-related targets has emerged, enabling simultaneous targeting of epigenetic and metabolic pathways.

**Figure 6:**
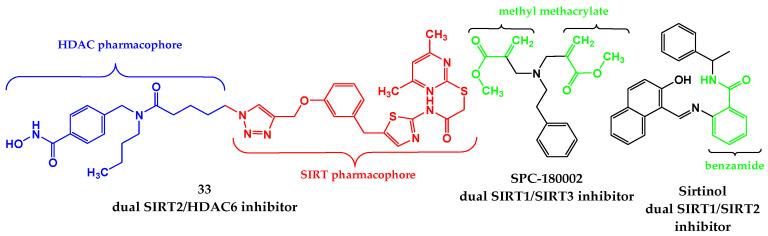
Chemical structures of representative dual inhibitors of SIRT and other targets. HDAC pharmacophore is colored blue and SIRT pharmacophore is colored red. Scaffolds related to inhibitory activity are colored in purple [130,131,132].

**Figure 7:**
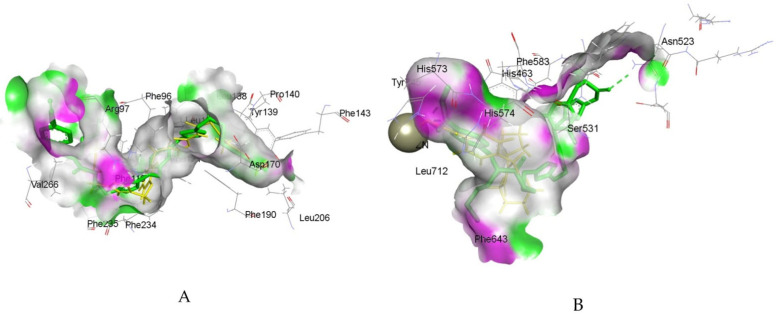
Visualization of the binding mode of compound **33** (dual SIRT2/HDAC6 inhibitor) in the active sites of (**A**) SIRT (PDB: 8OWZ) and (**B**) HDAC (PDB: 8G20). Compound **33** is represented with green sticks, while reference ligands are represented with yellow sticks.

The first-in-class dual SIRT2/HDAC6 inhibitors were developed by Sinatra et al. [130]. To design highly interesting new tools for dual inhibition of tubulin deacetylation, strategies of merging or linking the SIRT2- and HDAC6- pharmacophores were applied. A hydroxamic acid moiety from HDAC inhibitors was directly connected to the benzyl moiety of SirReal1. The pharmacophore linking approach incorporated a triazole-based linker to bridge the SirReal-based pharmacophore and the N-hydroxybenzamide. In biochemical in vitro assays, compound Mz325 (**33**) (Figure 6) was identified as a potent and selective inhibitor of both target enzymes (IC_50_ (SIRT2) = 0.32 μM and IC_50_(HDAC6) = 0.042 μM). Molecular docking of derivative **33**, performed using the Schrödinger software version 2021.3v, showed that both the HDAC6 inhibitor part and the SIRT2 inhibitor part bind to the corresponding target in a similar mode as observed for the reference unconjugated ligands in the new cocrystal structures (SIRT2 (PDB: 8OWZ) and HDAC6 (PDB: 8G20)). The binding mode of **33** overlaid with reference ligands in the active sites of SIRT and HDAC, obtained using GOLD 2022.3.0 Software [120] and visualized in Discovery Studio software v24.1.0.23298 [121], is shown in Figure 7.

To investigate the requirement for combined targeting of both SIRT1 and SIRT2 to induce p53 acetylation and cell death, three SIRT inhibitors, a benzamide derivative, Sirtinol [133] (Figure 6), a phenylpropanamide derivative, Salermide [134], and a carboxamide derivative, EX527 [135], were investigated for dual SIRT1/SIRT2 inhibitory action by Peck et al. [131]. In vitro assays regarding human recombinant Sirt1 and Sirt2 using a synthetic p53-derived peptide as a substrate showed inhibitory activities for Sirtinol (IC_50_ (SIRT1) = 37.6 μM and IC_50_ (SIRT2) = 103.4 μM), Salermide (IC_50_ (SIRT1) = 76.2 μM and IC_50_(SIRT2) = 45.0 μM), and EX527 (IC_50_ (SIRT1) = 0.38 μM and IC_50_(SIRT2) = 32.6 μM). For molecular docking, the LibDock algorithm from the Discovery Studio package (version 2.1) was used. In docking simulations (SIRT1, PDB: 2HJH and SIRT2, PDB: 1J8F), Sirtinol and Salermide expressed high degrees of selectivity for SIRT1/2, whereas EX527 was selective for SIRT1 but not SIRT2. Amino acid residues recognized as crucial for inhibitor binding are Phe 273, Phe 297, Gln 345, and His 363 of SIRT1 and Phe 96, Phe 119, Gln 167, and His 187 of SIRT2. It was shown that Sirtinol and Saleramid establish hydrogen bonds with Gln 345 and His 363 of Sirt1, while EX527 formed a hydrogen bond with Gln 345, with additional hydrophobic interactions with His 363 and Phe 273. On the other hand, Sirtinol and Saleramide form comparable hydrogen bonds with Gln 167 of SIRT2, while for EX527, any significant interactions with SIRT2 were not observed. Overall, the in vitro and docking results together indicated that simultaneous inhibition of both SIRT1 and SIRT2 is required to induce cell death, whereas EX527, targeting SIRT1 alone, induced cell cycle arrest at G1.

Considering that a methyl methacrylate group may be critical for SIRT inhibition, Cho et al. performed in-house chemical library screening [132]. Compound **SPC-180002** (Figure 6), selected for further in vitro SIRT activity assays, exhibited significant potency against SIRT1 (IC_50_ = 1.13 μM) and SIRT3 (IC_50_ = 5.41 μM). AutoDock Vina (version 1.1) was employed for molecular docking studies. The results suggested essential interactions with amino acid residues in the active site of SIRT1 (PDB: 4I5I) and SIRT3 (PDB: 4BV3). Hydrophobic interactions with Phe297, Phe293, Phe413, Ile411, Ile316, and Ile270 were observed in the binding place of SIRT1, as well as hydrophobic interactions with Phe180, Phe157, Ile230, Ile154, and His248 and the pyridine moiety of the cofactor NAD+ in SIRT3. In addition, the carbonyl group of **SPC-180002** formed hydrogen bonds with Ile230 and Asp231 in the binding pocket of SIRT3. The overall outcome indicated that **SPC-180002** is a promising lead compound with potential to be developed as a novel anticancer drug.

### 4.5. Dual Inhibitors of VEGFR-2 and Other Cancer-Related Targets

The design of dual inhibitors targeting VEGFR-2 and other cancer-related targets is an increasingly important strategy to combat cancer on multiple fronts, including angiogenesis and proliferation, while balancing efficacy and toxicity. The clinical relevance of this approach is already evident in approved drugs such as Lenvatinib and Cabozantinib, which outperform single-target therapies in various malignancies [136,137].

**Figure 8:**
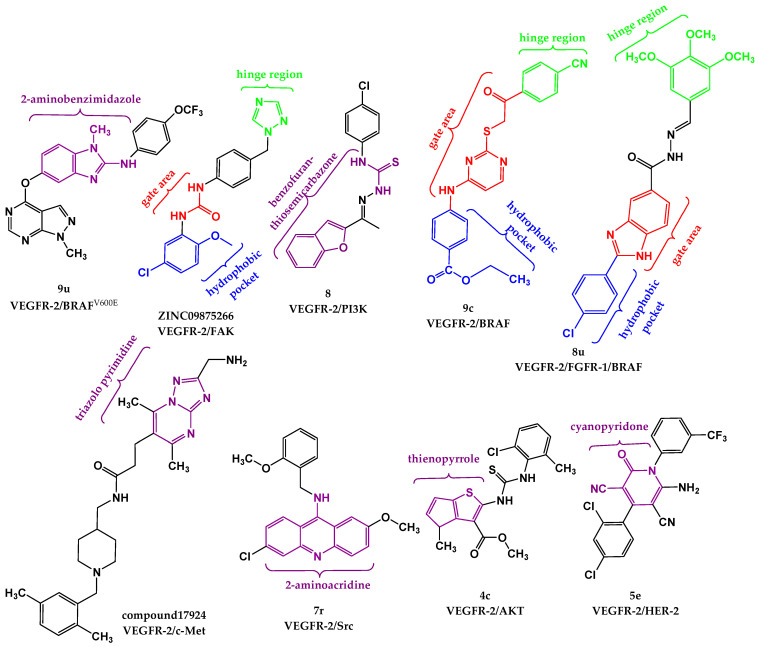
Chemical structures of representative dual inhibitors of VEGFR and other cancer-related targets. Key structural features for interaction with type II kinase binding sites, alosteric hydrophobic pocket, gate area, and hinge region, are colored in blue, red, and green, respectively. Other pharmacophores and scaffolds for the design of new derivatives are colored purple [139,140,141,142,143,144,145,146].

A series of 1H-pyrazolo[3,4-d]pyrimidine derivatives were designed as dual VEGFR-2/BRAF^V600E^ inhibitors [138] by Wang et al. Cyclization of a previously designed pan-RAF inhibitor to the corresponding 2-aminobenzimidazole derivative was carried out to improve conformational rigidity, while retaining the ability to form hydrogen bonds, which are required for activity. Compound **9u** (Figure 8) expressed high kinase inhibitory activity (VEGFR-2 (IC_50_ = 0.779 μM) and BRAF^V600E^ (IC_50_ = 0.171 μM)) and in vitro anti-proliferative effects, as well as a very good selectivity profile over 17 various protein kinases. Molecular docking procedures were performed using Glide (Grid-based Ligand Docking with Energetics) in Extra-Precision (XP) mode. Docking results in the active sites of VEGFR-2 (PDB: 3WZE) and BRAF^V600E^ (PDB: 1UWJ) showed that all representative compounds could adopt interaction patterns similar to reference ligands, including the formation of key hydrogen bonds with Cys919 of VEGFR-2 and with Cys532 of BRAF^V600E^.

The discovery of drugs with dual effects on VEGFR-2/FAK for potential use in cancer treatment was conducted by Fouad et al., who reported a significant correlation between VEGFR-2 and FAK signaling and their impact on tumor development and angiogenesis [139]. Initially, the structural resemblance of VEGFR-2 and FAK was examined using 3D alignment of VEGFR-2 (PDB ID: 4ASD) and FAK (PDB ID: 4K9Y), specifically analysing the overlap of amino acids in the binding site. In their chemical structure, type II kinase inhibitors comprise three structural features required for activity, designated as the “allosteric hydrophobic pocket”, the “gate area” (binding to Asp1046 and Glu885 of VEGFR-2 and to Asp564 and Glu471 of FAK) and the “hinge region” (binding to Cys919 of VEGFR-2 and Cys502 of FAK) [139]. Subsequent to the screening of the ZINC database, which consolidates data that can be bought or accessed with a licence, a molecular docking study was performed using Molecular Operating Environment (MOE, 2020.0901) software, providing the recognition of 13 compounds that satisfy all necessary interactions with the VEGFR-2 and FAK kinase regions. The lead (ZINC09875266) was selected for additional investigations of dual inhibitors (Figure 8, “alosteric hydrophobic pocket”, blue; “gate area”, red; and “hinge region”, green).

A structural hybridization strategy was applied to design 4-amino-2-thiopyrimidines as dual VEGFR-2/BRAF kinase inhibitors by combining 4-substituted aminopyrimidines (VEGFR-2 inhibitors) and 2-thioxopyrimidines (BRAF inhibitors) as antiangiogenic and antiproliferative agents [140]. Compound **9c** (Figure 8, “allosteric hydrophobic pocket”, blue; “gate area”, red; and “hinge region”, green) demonstrated promising dual kinase inhibitory activity on VEGFR-2 (IC_50_ = 0.17 mM) and BRAF (IC50 = 0.15 mM). Molecular docking procedures were done using Molecular Operating Environment (MOE, 2010.10) software. Docking results of the created compounds in VEGFR-2 (PDB ID: 4ASD) and BRAF (PDB ID: 1UWH) active sites revealed hydrogen bond interactions of **9c** with Glu885 and Asp1046 of VEGFR-2 and with Glu500 of BRAF, which are indispensable in the binding sites of both kinases.

**Figure 9:**
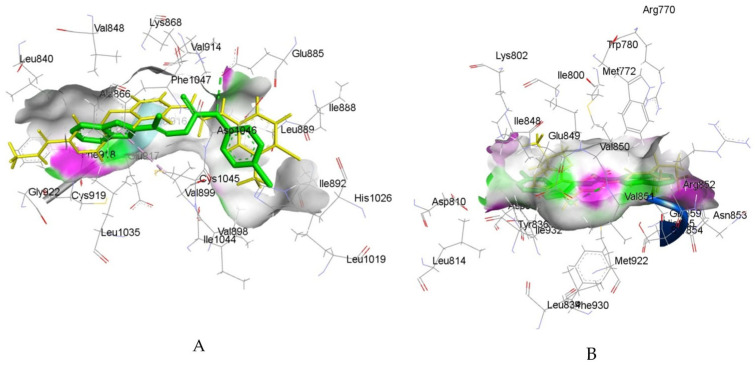
Visualization of the binding mode of compound **8** (dual VEGFR-2/PI3K inhibitor) in the active sites of (**A**) VEGFR-2 (PDB: 3WZE) and (**B**) PI3K (PDB: 4JPS). Compound **8** is represented with green sticks, while the reference ligands are represented with yellow sticks.

El-Khouly et al. implemented molecular hybridization rational design to discover new benzofuran derivatives (benzofuran–piperidine, benzofuran–piperazine, benzofuran–thiosemicarbazone, benzofuran–semicarbazone and benzofuran–benzylidine amide hybrids) as potential dual VEGFR-2/PI3K inhibitors [141]. Benzofuranyl thiosemicarbazone derivative, compound **8** (Figure 8), demonstrated activity against VEGFR-2 (IC50 = 68 nM) and PI3K (IC50 = 2.21 nM) as well as pronounced effects against hepatocellular and cervical cancer cell lines. To support the biological evaluation findings, molecular docking simulations were performed using the Molecular Operating Environment (MOE) program with crystal structures of VEGFR-2 with sorafenib (PDB: 3WZE) and PI3Kα with alpelisib (PDB: 4JPS). Important hydrogen bond forming was observed between compound **8** and Val851, Lys802, Arg770 and Gln859 of PI3Kα and Glu885, Cys1045 and Asp1046 of VEGFR-2. Therefore, compound **8** was highlighted as the most pronounced for later enhancement in targeting hepatocellular carcinoma and cervical cancer. The docked interaction mode of compound 8, consistent with reference ligands in the active site of VEGFR-2 and PI3K, obtained using GOLD 2022.3.0 Software [120] and visualized in Discovery Studio software v24.1.0.23298 [121], is shown in Figure 9.

Considering the acridine scaffold as a known core of topoisomerase inhibitors, Luan et al. designed 9-aminoacridine derivatives as dual VEGFR-2/Src inhibitors without inhibiting topoisomerase [142]. Virtual screening methods were used to screen PubChem and in-house compound libraries, leading to recognition of two compounds with an acridine scaffold and dual VEGFR-2 and Src inhibitory activity. Compound **7r** (Figure 8) expressed VEGFR-2 and Src inhibition of 44% and 8% at 50 μM, respectively, without topoisomerase inhibition. Molecular docking protocols, in Discovery Studio.2.5/Libdock, were performed for **7r** using the crystal structures of VEGFR-2 with 2-fluoro-5-trifluoromethyl phenyl-urea (PDB: 1YWN) and Src (PDB: 2H8H). Important hydrogen bonds were noticed with amino acids Cys917 of VEGFR-2 and Met341 of Src, as well as hydrophobic interactions with Lys866, Val914 and Leu838 of VEGFR-2 and Ser345 of Src. The observed binding mode indicated that lead compound **7r** adopts a compatible binding orientation and can interact with the essential amino acid residues of the VEGFR-2 and Src kinase binding pockets, thus may be investigated further as a potent VEGFR-2/Src dual inhibitor.

Implementation of a pharmacophore fusion strategy contributed to the development of dual VEGFR-2/AKT inhibitors [143]. By merging thienopyrrole and pyrrolothienopyrimidine scaffolds into one molecule, novel thiophene derivatives with potential antiproliferative activity were created. The most promising leads based on kinase inhibitory activity were compounds **4c** (IC_50_(VEGFR-2) 0.075 μM and IC_50_(AKT) = 4.60 μM) and **3b** (IC_50_(VEGFR-2) = 0.126 μM and IC_50_(AKT) = 6.96 μM). Molecular docking protocols were achieved using Discovery Studio 4.1 software (Accelrys, Inc., San Diego, CA, USA). Docking simulations that justified the interaction pattern of the docked compounds were similar to those of the reference ligands inside the active sites of both VEGFR-2 (PDB: 3EWH) and AKT (PDB: 4EJN). Hydrogen bonds considered important for inhibitory activity are with Thr916, Leu840 and Cys919 of VEGFR-2 and Gln79 and Tyr272 of AKT. Additionally, hydrophobic interactions with Ala866 and Cys1045 of VEGFR-2 and with Trp80, Arg273 and Ile84 of AKT contributed to the stabilization of the ligand complexes with the active sites.

Based on the cyanopyridone scaffold, a series of non-fused and fused compounds were constructed, including derivatives of 6-amino-1,2-dihydropyridine-3,5-dicarbonitrile and 3,4,7,8-tetrahydropyrimidine-6-carbonitrile [144]. Given the known antiproliferative activity of cyanopyridones and pyridopyrimidine-based anticancer molecules, the synthesized analogues were investigated as dual VEGFR-2/HER-2 inhibitors. The highest antiproliferative activities were observed for compounds **5a** (Figure 8) and **5e**, which were subjected to in vitro kinase assays against VEGFR-2 (IC_50_(5a) = 0.217 μM and IC_50_(5e) = 0.124 μM) and HER-2 (IC_50_(5a) = 0.168 μM and IC_50_(5e) = 0.077 μM). Molecular Operating Environment (MOE) 2019.02 was employed for molecular docking procedures. Docking of compounds **5a** and **5e** into VEGFR-2 (PDB: 4ASD) and HER-2 (PDB: 3RCD) active sites showed desired values and binding modes that are in agreement with in vitro estimations. Required interactions included hydrogen bonds with the amino acid residues Cys1045, Asp1046, Phe1047, Val898, Ile1044 and Glu885 were observed in the active site of VEGFR-2, as well as with Glu770, Ile767, Ala771, Val797, Thr798, Lys753, and Asp863 residues of HER-2.

Taking into consideration the synergistic role of VEGFR-2 and mesenchymal–epithelial transition factor (c-Met) in angiogenesis and tumor progression, dual VEGFR-2/c-Met inhibitors were investigated by virtual screening and molecular docking [145]. In accordance with the properties of the receptor-ligand complex, models of pharmacophores were defined, which enabled later screening of the ChemDiv database. The crystal structures of VEGFR-2 (PDB: 2OH4) with GIG as a reference ligand and c-Met (PDB: 3EFK) in complex with MT4 were used for molecular docking to recognize derivatives with strong binding affinities. Docking procedures were based on the application of both Libdock and CDOCKER modules within BIOVIA Discovery Studio 2019 (Dassault Systèmes BIOVIA, San Diego, CA, USA). Potential candidates as promising VEGFR-2/c-Met dual inhibitors, **compound17924** (Figure 8) and **compound4312,** were identified. Hydrogen bonds with amino acid residues Glu883, Asp1044, and Ile1023 and hydrophobic interactions with Cys1043, Val912, and Val897 in the active site of VEGFR-2, as well as hydrogen bonds with the residues Tyr1159, Met1160, and Lys1161 and hydrophobic interactions with Lys1110, Phe1124, Gly1128, Ile1130, Leu1140, Leu1142, Val1155, and Ala1221 in the c-Met active site, appeared to be the indispensable interactions required for action.

A pharmacophore modeling approach through virtual screening of an in-house scaffold of consolidated data provided recognition of a benzimidazole-based scaffold which was further structurally optimized to design derivatives as novel VEGFR-2/FGFR-1/BRAF multi-kinase inhibitors [146]. Molecular docking simulations were performed using Molecular Operating Environment (MOE, 2022.02) software with VEGFR-2 (PDB: 4ASD), FGFR-1 (PDB: 4V01) and BRAF (PDB: 5CT7). Interaction patterns of the designed compounds analogous to co-crystallized ligands were observed, interacting with key amino acids in the active sites VEGFR-2 (Glu885, Cys919 and Asp1046), FGFR-1 (Glu531, Ala564 and Asp641) and BRAF (Glu501, Cys532 and Asp594). After biological assays of multi-kinase inhibitory activity, compound **8u** (Figure 8, “alosteric hydrophobic pocket”, blue; “gate area”, red; and “hinge region”, green) emerged as a lead with strong inhibitory activity for VEGFR-2 (IC_50_ = 0.93 μM), FGFR-1 (IC_50_ = 3.74 μM) and BRAF (IC_50_ = 0.25 μM). Additionally, testing of compound **8u** revealed effects on cell cycle arrest and cell death [146].

## 5. Overall Evaluation of In Silico Approaches Targeting VEGFR/SIRT Enzymes and Future Perspectives

The outcomes of QSAR studies on VEGFR-2 inhibitors [62,63,64,65,66,67,68,69,70,71,72,73,74,75,76,77,78,79,80,81,82,83,84] analyzed in this review unequivocally indicate continuous advancement in modelling techniques, progressing from early MLR and CoMFA/CoMSIA to more sophisticated machine learning (SVM, ANN, RF, GBT) and hybrid approaches. Both groups of methods provide mechanistic insight into molecular interactions. Early MLR models [62,63] emphasize the contributions of hydrophobic, steric, and electronic interactions, while later machine learning studies reveal the central role of specific structural features such as hydrogen bonding [79], electrostatics [77], and heteroatoms like nitrogen [81] in determining interaction patterns. Methodologically, nonlinear machine learning approaches (e.g., LS-SVMs, GA-SVR, ANN) [64,70,73,77,80] have consistently surpassed traditional regression in predictive power, highlighting the growing importance of complex descriptor-activity relationships. Mechanistically, 3D QSAR contour mapping has often shown that steric bulk, electrostatic complementarity, and hydrogen bond positioning play key roles in activity [69,71,72,74]. Substituent effects across multiple scaffolds (pyrazine, benzazepinone, quinoxaline, thiourea) show consistent trends: bulky groups at specific positions, electron-withdrawing substituents, and heteroatoms capable of donating electrons enhance VEGFR-2 binding [69,71,74,79,81]. Larger datasets [80,83] have enabled the integration of fingerprints, docking, and dynamics, bridging ligand- and structure-based design. More recent models [75,84] are more likely to employ hybrid approaches, further combining genetic algorithms, Topomer CoMFA, and Monte Carlo methods to identify novel inhibitory scaffolds. Overall, these studies confirm that VEGFR-2 inhibition is driven by a synergy of intermolecular noncovalent interactions, with machine learning providing robust prediction methods. Methodological developments are ongoing, increasingly emphasizing the integration of different computational strategies and multiple descriptors for the discovery of anti-angiogenesis drugs.

Available QSAR studies on SIRT inhibitors [85,86,87,88,89,90,91,92,93,94,95,96,97,98,99,100,101,102,103,104] indicate a significant methodological shift, moving from 3D-QSAR models (CoMFA/CoMSIA) [85,86,87,88] on small datasets to integrated approaches that combine pharmacophore modeling, SMILES-based descriptors [90,91,93], and advanced machine learning techniques [100,101,103,104,105]. For SIRT1 inhibitors, steric, electrostatic, and hydrophobic interactions, as well as nitrogen and oxygen fragment contributions, have consistently emerged as the most significant features, while studies of activators have focused on atomic charges and electronegativity [85,86,87,88,89,90,96,97,104]. Analysis of the SIRT2 protein has revealed the hydrophobic character of its binding pocket, the importance of meta-substituted aromatic compounds, halogen interactions, and specifically positioned hydrogen bond-forming groups [88,92,95,98,99,100,101,102,103,104,105]. Larger datasets have contributed to robust machine learning QSAR models, where nonlinear methods (SVM, RF, GBT, kNN) outperform multilinear regression, improving prediction accuracy and supporting virtual screening and drug repurposing. FDA-approved drugs and peptide inhibitors have also been evaluated, expanding the applicability of these models [99,100]. Integrated 2D/3D QSAR and pharmacophore analyses have shown that structural motifs such as amide donors, pyridine nitrogen atoms, and hydrophobic groups are critical determinants [94]. Isoform selectivity remains a recurring challenge, with newer models incorporating SIRT1/2 and SIRT2/3 activity. The research field has evolved from scaffold-restricted SAR to generalizable predictive pipelines. Overall, these studies emphasize the importance of combining ligand- and structure-based strategies, nonlinear machine learning methods, and diverse chemical datasets to advance the rational design of selective SIRT modulators.

As can be seen from Table 1, most of the QSAR models for VEGFR-2 inhibitors are related to small, specific and homogeneous datasets, which have advantages for future predictions of specific types of pharmacophores. Small datasets have high chemical consistency and better interpretability, which can indicate stricter SAR rules. In several cases [70,79], more diverse datasets were used for more general predictions covering different but important pharmacophores for VEGFR-2 inhibition. More general models have advantages in predicting activity for a broad number of scaffolds and can be used for virtual screening purposes to predict a wider biochemical space. In almost all models, the dataset was divided into a training and validation set, ensuring the robustness and predictivity of the QSAR models. The results ensured robust and predictive performance, especially with validation parameters as an external predictivity measure. In rare cases, e.g., [62], external validation was not provided. For more detailed insight into statistical parameters, readers are referred to the original publications listed in Table 1.

As can be seen from Table 2, QSAR models for SIRT1-3 are also related to both specific and more general chemical space. More general models are reported, e.g., [87,91,93,102,103,105] for SIRT1-3 inhibition and activation, while specific datasets are reported in e.g., [85,86,88,95,98,101,104,106]. As we stated before, more general models have advantages in predicting activity for a variety of scaffolds and quantification purposes, while small models are better for mechanistic understanding of specific chemical types, and with high-quality data with consistency in biological assays.

In all models, the dataset was divided into training and validation sets, providing robustness and predictive reliability. In general models, like those from, e.g., Worachartcheewan et al. [91], and Djokovic et al. [103], in-depth preparative and statistical analysis were provided.

For more detailed insight into statistical parameters, readers are referred to the original publications listed in Table 2.

In most cases, data curation was not provided for either the VEGFR2 or SIRT QSAR models. Exceptions include in-depth analyses in the general VEGFR2 model by Tripathi et al. [80], Worachartcheewan et al. [91], and Djokovic et al. [103] for SIRT inhibitors. However, chemicals in small datasets usually come from the original sources from the same laboratory or research groups and cleaner data and data curation are already provided. Experimental noise and imbalance in quality regarding different assay protocols are reduced. In more diverse datasets, data curation was provided in examples, e.g., [80,91,92,96,103]. For better comparative insight about dataset size and chemical types, please see Table 1 and Table 2. The applicability domain can be defined based on the consistent scaffold within the dataset, although the authors themselves mainly did not emphasize this in the articles. Regarding the applicability domain, most small datasets contain highly consistent chemical structures related to a specific scaffold, which can partially bypass extrapolation, considering that the model will be used exclusively for a similar chemical space. General models cover diverse chemical space, but far from all possible chemicals. For more general models, the applicability domain was provided in models, e.g., [82,83,90,91,92,93,102,103,104], while in some cases information is missing, e.g., [87,99,100,105]. The applicability domain will give confidence regarding different scaffolds’ prediction and extrapolation, but it may also offer insight into unreliable predictions caused by assay heterogeneity, which is often present in large datasets extracted from available libraries of bioactive molecules.

Strategic use of SBDD can be considered in the development of dual and multi-target inhibitors, particularly VEGFR/HDAC, VEGFR/EGFR, VEGFR with other targets (BRAF, FAK, PI3K, Src, AKT, HER-2, c-Met, FGFR-1), and dual SIRT/HDAC inhibitors [116,117,118,126,127,128,129,138,139,140,141,142,143,144,145,146]. Available articles on dual VEGFR/HDAC inhibitors describe rational efforts to combine pharmacophores for epigenetic and angiogenesis targeting, with pazopanib-derived hybrids, quinazoline analogues, and benzofuran derivatives showing significant dual binding affinities, validated by docking to the active sites of VEGFR-2 and HDAC [116,117,118]. Reviewed research highlights the importance of integrating zinc-binding groups (hydroxamates, ortho-aminoanilides) with kinase pharmacophores, enabling low IC_50_ values, though concerns remain regarding the balance of selectivity and toxicity. Similarly, structural similarities have been observed between dual VEGFR/EGFR inhibitors and the ATP-binding sites of receptor tyrosine kinases [126,127,128,129]. However, despite significant in vitro evaluation data confirming activity, the risk of off-target effects and systemic toxicity remains a critical obstacle to implementing this strategy. The design of other VEGFR dual inhibitors (VEGFR/BRAF, VEGFR/FAK, VEGFR/PI3K, VEGFR/Src, VEGFR/AKT, VEGFR/HER2, VEGFR/c-Met, and VEGFR/FGFR-1/BRAF) indicates a clear trend toward polypharmacology, supported by the implementation of pharmacophore fusion and hybridization strategies [139,140,141,142,143,144,145,146]. Strategies involving synergies may help reduce drug resistance, but optimization becomes more complex as accurate binding alignment to multiple targets must be achieved. In parallel, the design of SIRT-based dual inhibitors (SIRT/HDAC, SIRT1/2 combinations) serves as a significant example of how epigenetic drug design can integrate various computational techniques (pharmacophore fusion and molecular docking) to optimize binding interactions while maintaining isoform selectivity [130,134,135].

Dual-target inhibitors present specific challenges, including the risk of off-target interactions, potential toxicity, and complex pharmacokinetic behavior. Overcoming these issues requires rigorous molecular design to improve selectivity, systematic assessment of dose–response relationships, and early consideration of pharmacokinetic and ADMET profiling. In all the articles reviewed, molecular docking and pharmacophore combination strategies validated binding modes using specific crystallographic data, but most studies remain preclinical and rely only on in silico estimations. The main cause of this discrepancy is the limited research on ADMET characteristics and pharmacokinetic optimization, which are necessary to advance these leads toward clinical trials. Overall, available studies illustrate both the promising potential and complexity of SBDD-guided multi-target cancer therapeutics. They demonstrate advances in rational pharmacophore combination but also highlight ongoing challenges in achieving target selectivity, minimizing toxicity, and ensuring clinical relevance. Despite clear methodological progress and valuable insights provided by CADD approaches, the predictive reliability of in silico findings remains limited by model assumptions, dataset quality, and the complexity of tumor biology. Most in silico-designed dual VEGFR/SIRT inhibitors, as well as other multi-target anticancer agents, have not yet progressed to experimental or preclinical validation, and critical parameters such as ADMET properties and off-target effects are often insufficiently characterized at early stages. Therefore, while CADD methods are valuable for efficiently identifying promising scaffolds and selecting candidates for synthesis, integration with rigorous in vitro and in vivo experimental testing is necessary to confirm biological efficacy and safety before clinical translation.

The integration of advanced computational techniques in CADD with experimental research holds promise for developing highly specific and potent dual-target inhibitors that simultaneously modulate VEGFR and epigenetic signaling pathways. As our knowledge of the complex molecular processes underlying carcinogenesis grows, personalized therapeutic strategies targeting these interconnected mechanisms will likely become more feasible, potentially leading to improved patient outcomes. Available preclinical studies of dual VEGFR/epigenetic inhibitors, including pazopanib-derived HDAC/VEGFR-2 analogs [118], have shown improved antiproliferative and antiangiogenic activity in cellular and xenograft models compared to single-target approaches. Although clinical validation is still lacking, these findings provide an initial experimental basis for the therapeutic potential of simultaneous modulation of angiogenic and epigenetic pathways. Greater appreciation of the biological approach may provide deeper insight into the mechanisms of crosstalk and resistance, contributing to the development of multitargeted therapies. Furthermore, the development of more sophisticated in silico techniques, including molecular modeling and virtual screening, is expected to enhance the estimation of drug-target interactions and pharmacokinetic properties, thereby streamlining the preclinical validation process. As the importance of in silico studies continues to grow and open-access datasets expand, interdisciplinary collaborative efforts are needed to accelerate the translation of computational discoveries into clinical applications, with the ultimate goal of providing safer and more effective cancer treatments targeting both the VEGFR and SIRT pathways. An excellent example that could serve as a guide for future perspectives is the successful implementation of an in silico methodology in the identification of the compound GW856804X as a potent SIRT2 inhibitor [147]. This compound was originally reported as a VEGFR-2 inhibitor [148], but the application of computational techniques, including virtual screening and docking, revealed its potential to target SIRT2 [147]. In the aforementioned study, VEGFR-2 inhibition was not experimentally examined, but computational and biochemical data confirmed the applicability of integrated in silico strategies for the potential repurposing of available kinase inhibitors toward epigenetic targets such as SIRT2 [147]. Therefore, this study highlights the growing significance of in silico methodology as indispensable in the discovery of new anticancer agents. Emerging technologies such as AI-driven drug design and multi-omics data integration may further refine the process of selecting dual VEGFR/SIRT inhibitors. In contrast to conventional QSAR and classical CADD approaches, which rely largely on predefined molecular descriptors and structural similarity, AI-based models can process large-scale heterogeneous datasets and detect complex nonlinear relationships between molecular features and biological responses. Incorporating transcriptomic, proteomic, and metabolomic information may further support modeling with specific molecular backgrounds across different tumor types. These advances could improve the selection of dual-target anticancer agents with balanced selectivity and pharmacokinetic properties and help bridge the gap between in silico estimations and experimental findings. Future research should prioritize systematic experimental validation of dual VEGFR/SIRT inhibitors, designed and selected by in silico methods, through well-designed in vitro and in vivo studies, including comprehensive pharmacokinetic and toxicity assessments. Integrating medicinal chemistry with molecular modeling will be essential to optimize binding selectivity, potency, and overall drug-like properties. Further investigation of the interplay between VEGFR signaling and diverse epigenetic regulators across different cancer types may help refine target combinations and enhance translational relevance. Continued convergence of CADD, medicinal chemistry, molecular biology, and clinical research will be crucial for advancing dual-target strategies toward clinically effective anticancer therapies.

## 6. Conclusions

The complexity of cancer pathogenesis involves the dysregulation of multiple signaling pathways, notably VEGFR and SIRT, which are critical mediators of angiogenesis, cell survival, and proliferation. Simultaneously targeting these pathways offers a promising strategy for developing more effective and personalized anticancer therapies. Computational approaches, including QSAR modeling, molecular docking, virtual screening, and machine learning techniques, have significantly advanced the identification and optimization of novel inhibitors that can modulate VEGFR and SIRT activities. Drug discovery is complex and challenging, but the expanded use of an optimal combination of advanced in silico approaches has significantly increased the success rate in lead compound discovery. The ongoing exploration of complex molecular processes in carcinogenesis and progression provides new information and highlights the need to closely integrate computational tools with experimental validation in the discovery of novel anticancer agents targeting VEGFR and SIRT signaling pathways.

## Figures and Tables

**Table 1:** Summary of machine learning-based QSAR approaches applied to VEGFR-2 inhibitors.

Chemical Data	Data Size	Model/Method	Type of Descriptor	Reference
N-Phenyl-N′-{4-(4-quinolyloxy)phenyl}urea derivatives	29	Multiple Regression Analysis (MRA)	2D (ClogP, steric, electronic)	[62]
Benzoxazole derivatives	36	Pseudoreceptor-based QSAR with MPSO	Pseudo-probes (empty, electrostatic, steric, hydrophobic, H-bond donor, receptor)	[63]
Pyrazine-pyridine biheteroaryl derivatives	32	MLR & LS-SVM (non-linear)	Constitutional, topological, geometrical, electrostatic, quantum-chemical	[64]
VEGFR tyrosine kinase inhibitors	82	3D-QSAR CoMSIA	3D (steric, electrostatic, hydrophobic, H-bond donor/acceptor)	[65]
Naphthalene and indazole derivatives	61	GA-SVR	3D & 2D (oxygen atoms, molecular polarizability, van der Waals volume, mass)	[66]
D-angulated benzazepinone derivatives	32	3D-QSAR CoMFA & CoMSIA	3D (steric, electrostatic, hydrophobic, H-bond)	[67]
Aminopyrazolopyridine urea derivatives	32	2D-QSAR	2D (N atom position, polarizable groups, aromatic rings, H-bond donors)	[68]
Arylphthalazines & 2-((1H-azol-1-yl)methyl)-N-arylbenzamides	53	3D-QSAR CoMFA & CoMSIA	3D (steric, electrostatic, hydrophobic, H-bond)	[69]
VEGFR-2 inhibitors	192	MLR, PLS, PC-ANN	2D & 3D	[70]
4-Aminopyrimidine-5-carbaldehyde oxime & N-phenyl-N′-{4-(4-quinolyloxy)phenyl}urea derivatives	81	3D-QSAR PHASE	3D (hydrophobic, H-bond, aromatic)	[71]
4-Aminopyrimidine-5-carbaldehyde oxime derivatives	32	GA-MLR & GA-SVM	2D	[73]
Tetrahydro-3H-imidazo [4,5-c]pyridine derivatives	36	3D-QSAR CoMFA & CoMSIA	3D (steric, electrostatic, hydrophobic, H-bond)	[74]
Furo [2,3-d]pyrimidine & thieno [2,3-d]pyrimidine derivatives	33	ANN & MLR	2D autocorrelation (RDF035u, Mor24v, EEig11r, ATS3v, G2s)	[75]
6-Amide-2-aryl benzoxazole/benzimidazole derivatives	44	HQSAR & Topomer 3D-CoMFA	2D & 3D (HL, FD, FS, contour maps)	[76]
Benzothiazole derivatives	22	Multilinear regression QSAR	2D & 3D (SMR_VSA4, SMR_VSA5, dipoleZ)	[77]
Quinoxaline derivatives	33	MLR with Genetic Function Algorithm	2D (SpMax8_Bhs, GATS5e, GATS3i, GATS8i, VR2_Dt)	[78]
Thiourea-based VEGFR-2 inhibitors	98	GFA-PLS, PLS-HQSAR, k-MCA, Bayesian classification	2D & fingerprints (SpMax8_Bhs, GATS5e, GATS3i, GATS8i, VR2_Dt)	[79]
VEGFR-2 inhibitors (structurally diverse)	3584	ML: LR, DT, RF, kNN, GBT	2D & fingerprint	[80]
Triazolopyrazine derivatives	23	3D-QSAR CoMFA & CoMSIA	3D (steric, electrostatic, hydrophobic, H-bond)	[81]
Structurally diverse VEGFR-2 inhibitors	37	2D-QSAR forward stepwise MLR	2D (electro-topological, atom types, topological distance, 2D autocorrelation)	[82]
Benzo-fused heteronuclear derivatives	118	QSAR Monte Carlo regression	2D SMILES & graph-based descriptors	[83]
Benzoxazole/benzimidazole derivatives	45	MLR QSAR (GFA)	2D (SpMax5_Bhp, ATS5v, AATSC7v, MATS5c)	[84]

**Table 2:** Summary of machine learning-based QSAR approaches applied to SIRT biochemical space.

Chemical Data	Data Size	Model/Method	Type of Descriptor	Reference
Imidazothiazole and oxazolopyridine derivatives	33	3D-QSAR COMFA & COMSIA	Electrostatic, steric	[85]
Acridinediones	18	3D-QSAR	Hydrophobic, non-polar	[86]
Substrate-based SIRT1 inhibitors	79	3D-QSAR COMFA	Contour maps, steric/electrostatic	[87]
2-anilinobenzamide derivatives	46	3D-QSAR COMFA + MD & docking	Steric, electrostatic	[88]
Indole, aurones, thioacetyl lysine, pyrimidine carboxamide, sirtinol derivatives	79	Energy-based pharmacophore + 3D-QSAR PLS	Functional group features	[89]
Various compounds (SMILES descriptors)	45	QSAR Monte Carlo linear regression	SMILES fragments	[90]
Various SIRT1 & SIRT2 inhibitors	SIRT1 310 SIRT2 345	QSAR Monte Carlo	SMILES fragments	[91]
SIRT2 inhibitors	96	MLR & kNN (GFA)	Pharmacophores, molecular descriptors	[92]
SIRT1 modulators	112 (67 activators + 45 inhibitors)	ML classification Monte Carlo	SMILES fragments	[93]
Imidazothiazole and oxazolopyridine derivatives	50	2D & 3D-QSAR	Steric, electrostatic, hydrophobic, H-bond acceptor	[94]
Thieno [3,2-d]pyrimidine-6-carboxamide derivatives	30	Pharmacophore + 3D-QSAR PLS	H-bond donors/acceptors, hydrophobic	[95]
SIRT1 activators	65	Hierarchic 3D-QSAR (HiT QSAR) PLS	Atomic charges, electronegativity, H-bonds, lipophilicity	[96]
Imidazothiazole, oxazolopyridine, azabenzimid scaffolds	30	QSAR MLR	Electronegativity, charge, polarizability, WHIM index	[97]
3′-Phenethyloxy-2-anilinobenzamide derivatives	75	2DHQSAR + 3D COMFA & COMSIA	Molecular fingerprints, 3D features	[98]
SIRT2 inhibitors, including SirReal2	39	Atom & Field-based 3D-QSAR PLS	Atom & field features	[99]
SIRT2 inhibitors	234	ML binary classification	Fingerprints	[100]
2-((4,6 dimethyl pyrimidine-2-yle) thio)-N-phenyl acetamide derivatives	33	MLR + SVR	2D & 3D descriptors (BELV2, GATS6e, GATS8p, RDF)	[101]
DEL dataset compounds	108,528 & 5,655,000	Regression QSAR	Not specified	[102]
SIRT2 inhibitors	1797	Regression & classification ML	Fingerprints & selected features	[103]
Nicotinamide-based SIRT2 inhibitors	86	3D-QSAR GRIND + ML classification	GRIND pharmacophores, docking features	[104]
Cyclic & non-cyclic SIRT2 peptide inhibitors	876	ML QSAR (RF, Ridge, GB, XGBoost)	Peptide descriptors	[105]
5-((3-amidobenzyl)oxy) nicotinamide compounds	64	3D-QSAR COMFA & COMSIA PLS	Electrostatic, steric	[106]

## Data Availability

No new data were created or analyzed in this study. Data sharing is not applicable.
